# Quaternary Ammonium Salts as Supporting Electrolytes in Cathodic Reductions: An Analysis of Their Electrochemical Stability

**DOI:** 10.1021/acs.jpcb.5c00650

**Published:** 2025-06-11

**Authors:** Florian Mast, Maximilian M. Hielscher, Eva Plut, Jürgen Gauss, Gregor Diezemann, Siegfried R. Waldvogel

**Affiliations:** † Department Chemie, Johannes Gutenberg-Universität Mainz, Duesbergweg 10-14, 55128 Mainz, Germany; ‡ Max-Planck-Institute for Chemical Energy Conversion, Stiftstrasse 34-36, 45470 Mülheim an der Ruhr, Germany

## Abstract

We present a study of the thermodynamic and kinetic electrochemical stability of a test set of more than 5000 singly charged and doubly charged cations containing a quaternary ammonium group. The redox potentials and the inner-sphere reorganization energies are calculated using density functional theory employing an implicit solvent model for the solvation. We find three different categories of cations regarding their behavior under one-electron reduction. One category fragmentizes upon reduction, in another category the nitrogen atom is part of a ring which opens upon electron addition and, finally, the systems of the third category containing multiple bonds undergo only minor structural changes. An important class of quaternary ammonium ions belonging to the first category is provided by the tetraalkylammonium ions that fragmentize into a tertiary amine and an alkyl radical. We observe that systems undergoing ring-opening appear to be somewhat more stable electrochemically than the other two categories on average. Experimentally, we, for example, find this behavior for a system containing the quaternary nitrogen atom as a member of a ring. We suggest that in addition to the routinely employed tetraalkylammonium ions also such systems are well suited for use as supporting electrolyte in electrochemical reductions. For all systems investigated, the adiabatic values for the electron affinities and the ionization potentials show strong linear correlations with the reduction potential and the oxidation potential, respectively. This, however, is not the case for the quantities computed from the frontier orbital energies of density functional theory. The inner-sphere reorganization energies are important quantities determining the activation energy of heterogeneous electron transfer and we find that the values calculated for the oxidized state and the reduced state are quite similar for all three categories of ions. For the cations containing multiple bonds the inner-sphere reorganization energies show a strong prevalence for very small values due to the strong structural similarity of equilibrium conformations in the oxidized and the reduced state. This finding is in accord with the known instability of systems containing a carbon–carbon double bond next to a charged nitrogen under acidic conditions. A simple model relating a single Huang–Rhys factor and an effective vibration frequency given by the sum of all mode frequencies to the inner-sphere reorganization energy works very well. The effective curvatures of the potential energy curves for the oxidized and the reduced cations are found to be almost identical, which we interpret as a hint toward the applicability of Marcus theory in the harmonic approximation to the electron transfer processes in quaternary ammonium cations.

## Graphical abstract

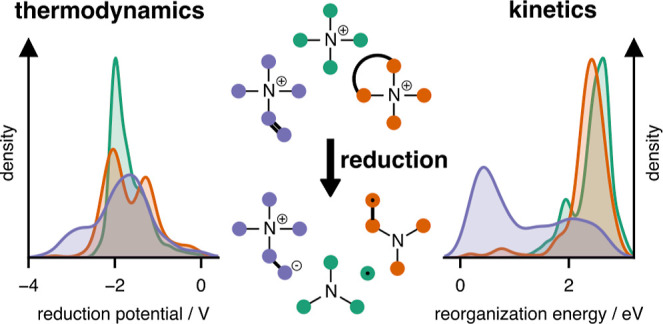

## 1 Introduction

Electrochemical reactions are among the most studied chemical reactions and they provide a broad field of applications. This gave rise to an enormously increasing interest into electrochemical synthesis during the past decade.^1–4^ Many examples of successful applications in the field of organic chemistry have been documented but for instance the experimental realization of cathodic reductions of amides to amines still are challenging.^5–7^

Reductions in aprotic media are known to be strongly influenced by the structure and the properties of the cations of the supporting electrolyte (SE).^8^ The SE has a number of different tasks to fulfill such as to protect the cathode material, take part in the structure of the double layer, increase the conductivity of the solution and it also might play a role as mediator in the electron transfer process.^9,10^

One class of SEs that is very often employed in electrochemical reaction studies is provided by quaternary ammonium salts and their properties have been the subject of a number of investigations.^11–15^ In particular, the study by Mousavi et al.^15^ revealed that structural aspects have only a limited influence on the electrochemical properties. In addition, for 18 systems a linear relation between the cathodic limit and the energies of the lowest unoccupied molecular orbital has been observed.^15^

When dealing with purely heterogeneous electron transfer,^16,17^ the impact of the SE cations is usually treated in an indirect manner, e.g. via the so-called Frumkin correction accounting for the interactions between the SE and the reactants in the double layer.^10,18^ However, in some situations, the cations of the SE might also act as mediator in the electron transfer process^19^ or give rise to (unwanted) side products. Therefore, it is important to study both, the thermodynamic and the kinetic electrochemical stability of quaternary ammonium cations (QACs) relative to the corresponding one of the reagents under consideration. The first step in the reduction of QACs of the NR_4_^+^-type often proceeds via the fragmentation of the cation into a tertiary amine and a radical^14^ but this is not necessarily the case. If the QAC does not only contain alkyl groups but also ring-like structures or multiple bonds different scenarios are feasible.

In the present paper we provide a computational study of properties related to the redox behavior and the electron transfer process of a large number of 5392 singly charged and doubly charged QACs that have been investigated earlier using a statistical analysis in order to relate their molecular, electric and electronic properties to the yield of cathodic reduction reactions assuming a purely heterogeneous electron transfer mechanism.^20^ All QACs were selected from the PubChem database^21^ and the set consists of systems with different chemical structures. It includes single-bonded systems, systems with multiple bonds and various functional groups like CO-, OH- and CN-groups and also ring-like structures. Additionally, we experimentally studied the electrochemical reduction of QACs containing the quaternary nitrogen atom as a member of a ring and three tetraalkylammonium ions.

The remaining organization of the present paper is as follows. In the next Section, we present the details of our calculations, followed by the presentation and discussion of our theoretical and experimental results. The paper closes with some conclusions in Section 4.

## 2 Computational Details

### 2.1 Data Set Determination and Electronic Structure Calculations

The actual set of QACs used in the present study is a subset of the ensemble created in ref 20. As explained in detail there, we used RDKit to search the PubChem database for QACs and used the iMTD-GC-algorithm^22,23^ as implemented in the program package CREST^22,24^ in order to perform a conformational search at the GFN2-xTB level of theory.^25^ Afterward, a geometry optimization with the ORCA program^26,27^ at the density functional theory (DFT) level employing the B97–3c-method^28^ was performed for the QACs and additionally for the oxidized and the reduced species of all QACs.

The same methodology was employed in all electronic structure calculations. For a subset of 100 QACs, we additionally performed all calculations using a method of higher quality and accordingly higher computational cost, see the discussion at the end of this Section.

Since in the present contribution the focus is on a discussion of the reduction and oxidation behavior of the ions, we refined the original data set. All structures that exhibited imaginary harmonic frequencies for the oxidized or reduced species were excluded. Filtering the data set in that way, a set of 5392 QACs remained.

For the computation of electronic and thermodynamic properties we started from the reference structure of the cations, the oxidized form, and the reduced forms as determined from the geometry optimization.

As explained below, for the determination of reorganization energies additional single-point energies were calculated for the whole data set. Using the computed harmonic frequencies internal energies, enthalpies, entropies and Gibbs free energies were obtained at a pressure of 1 atm and a temperature of 298.15 K.

Solvent effects were treated implicitly employing the COSMO implementation in TURBOMOLE.^29^ Using these results, the solvation energies in acetonitrile of all relevant oxidation states of the QACs were computed using the COSMOtherm software package^30^ and the COSMO-RS Fine method.^31^

#### 2.1.1 Molecular Dynamics Simulations

We applied MD simulations to justify our computational methodology for the calculation of various electronic properties based on the energy minimized structures.^20^ We started from the structures obtained from the geometry minimization and performed equilibrium MD simulations at room temperature and ambient pressure to sample the relevant conformations. We computed all features discussed in ref 20 for 4000 structures sampled from 400 ns simulations for each of 15 selected cations and compared them to the values obtained from the electronic structure calculations using the energy minimized structure, justifying our procedure.

In the present study, we used MD simulations to justify the method employed in the calculation of reorganization energies in case that the considered cations fragmentize. To this end, we estimated the time scale of separation of the products of the reduction of a tributyloctylamine cation into tributylamine and an octanyl radical in acetonitrile (MeCN) and in tetrahydrofuran (THF)^32^ with the result that the two subsystems can be viewed as one system for about 100 ps which is longer than the typical time scale of vibrational relaxation. In these simulations, we used a simulation box with a volume of 343 nm^3^ containing the fragments and either 3955 MeCN molecules or 2549 THF molecules. All simulations have been performed using the GROMACS simulation package^33,34^ employing the OPLS force-field.^35^

#### 2.1.2 Calculation of Redox Potentials

As described in ref 20 we calculated the standard redox potentials (RPs) using the solvation energies *G* ^solv^ of the cations and the reduced species in MeCN along with the vacuum free energies *G* ^g^ of the systems applying a Born–Haber cycle.^36^ Here, we apply the same methodology to compute the oxidation potentials (OPs). The scheme used in the calculations can be summarized as follows

1 ΔGXP=−1F(ΔGxg+ΔGxsolv)withΔGxg=Gg(Qnx+)−Gg(Q+)ΔGxsolv=Gsolv(Qnx+)−Gsolv(Q+)

for the reaction *x* corresponding to a reduction, x = red, *n*_red_ = 0 and Δ*G*_XP_ = Δ*G*_RP_ or an oxidation, x = ox, *n*_ox_ = 2 and Δ*G*_XP_ = – Δ*G*_OP_. The respective reactions are *Q* ^+^ + e^–^ → *Q* (with *Q* ^+^ denoting a singly or double charged ion) in case of a reduction and *Q* ^+^ → *Q* ^2+^ + e^–^ for an oxidation.

In the present calculations, we used the ferrocenium/ferrocene redox couple as the refs 37 and 38 . Using the same computational methodology as for the QACs, the reduction potential of this couple was found to be given by 4.83 eV and was subtracted. For comparison, the free energy change of a reference standard hydrogen electrode used in ref 20 is 4.44 eV.^36^ We mention that the accuracy of implicit solvent models for this type of calculations usually is high,^39^ although for some highly charged systems and in aqueous solution significant errors might occur.^40,41^

#### 2.1.3 Calculation of Reorganization Energies

The kinetics of electrochemical redox reactions usually is determined by thermally activated electron transfer processes.^42,43^ For heterogeneous electron transfer from the electrode to an ion usually the Marcus-Hush-Chidsey model for the transfer rate is applied and the activation energy in this case is determined mainly by the reorganization energy λ.^44–47^ The total reorganization energy is given by the sum of the inner-sphere reorganization energy, λ_*i*_, and the solvent (outer-sphere) reorganization energy, λ_out_ ^44,48–51^

2 $$\lambda =\lambda_{i}+\lambda_{out}$$

In a dielectric continuum model, for heterogeneous one-electron transfer the latter depends on the molecular radius *r*_mol_, the distance *R* to the electrode, the static dielectric constant ε_s_, and the one at high frequencies ε_∞_ ^44,48,51,52^

3 $$\lambda_{out}=\frac{e^{2}}{8\pi \epsilon_{0}}\left(\frac{1}{\epsilon_{\infty }}-\frac{1}{\epsilon_{s}}\right)\left(\frac{1}{r_{mol}}-\frac{1}{2R}\right)$$

where *e* is the elementary charge and ε_0_ denotes the permittivity of free space. The dielectric constants of MeCN at *T* = 298 K are assumed to be given by ε_∞_ ≃ 1.8 and ε_s_ ≃ 36.0.^49,53^

The inner-sphere reorganization energy is given by the mean of the reorganization energies λ_*i*_^ox red^ of the oxidized/reduced species^49^

4 $$\lambda_{i}=\frac{1}{2}\left(\lambda_{i}^{ox}+\lambda_{i}^{red}\right)$$

with

5 $$\lambda_{i}^{ox}=E_{ox}\left(R_{red}^{eq}\right)-E_{ox}\left(R_{ox}^{eq}\right)\;\mathrm{and}\;\lambda_{i}^{red}=E_{red}\left(R_{ox}^{eq}\right)-E_{red}\left(R_{red}^{eq}\right)$$

Here, *E*_ox_(*R*_red_^eq^) denotes the energy of the oxidized species at the equilibrium configuration *R*_red_^eq^ of the reduced species and similar for the other energies in the expression, cf. Figure 1. The energies occurring in eq 5 were computed in vacuum as single-point energies of the reduced species at the equilibrium geometry of the oxidized species and vice versa.^49,54^

**Figure 1:**
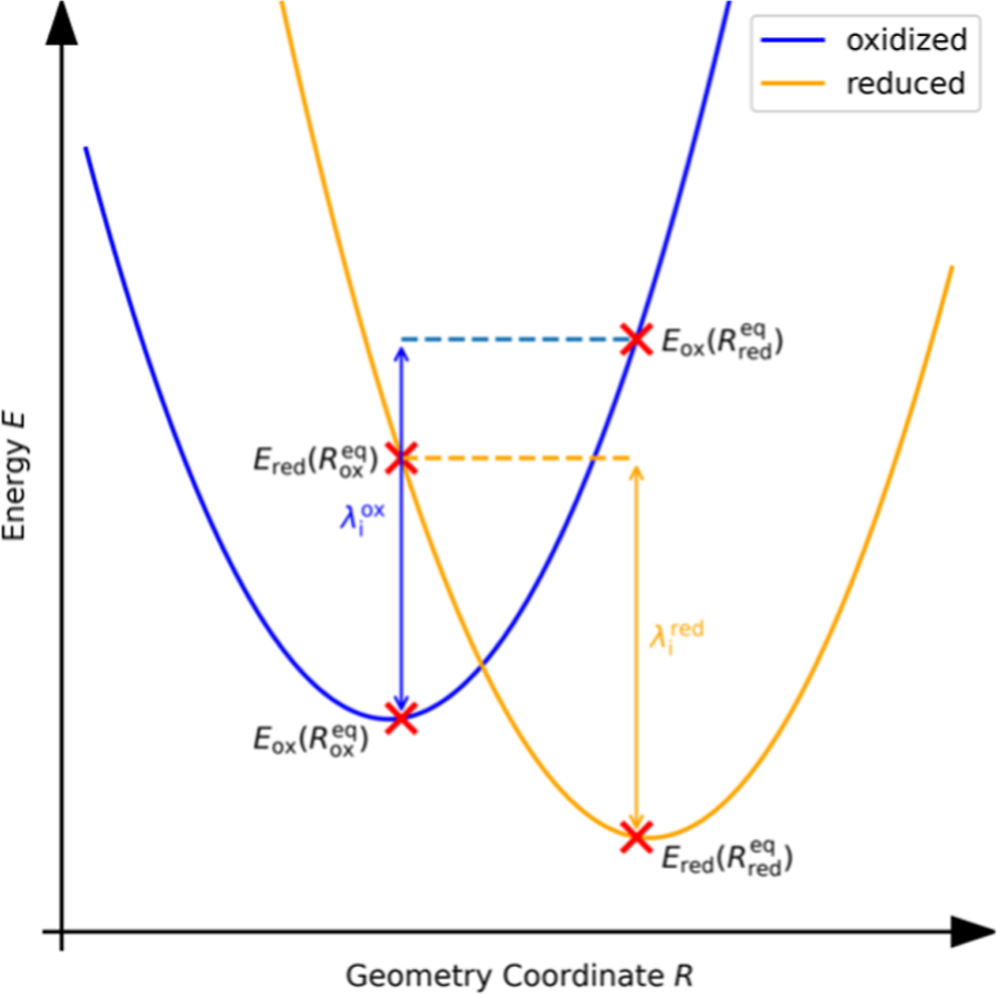
Definition of the various energies used in the calculation of the inner-sphere reorganization energies, eqs 4 and 5.

#### 2.1.4 Benchmark Calculations

Because the B97-3c-method is based on a GGA functional,^28^ which might not yield results of highest quality for redox potentials,^55^ we additionally performed DFT calculations using the range-separated hybrid, *meta*-GGA functional ωB97M-V^56^ in combination with a large triple-ζ basis set, def2-TZVPPD.^57,58^ Also these calculations were performed using ORCA and the same settings as for the B97-3c-calculations. Since these calculations are much more time-consuming, we were only able to perform benchmark calculations on a subset of our huge data set. In order to obtain results that are representative for our complete data set, we chose QACs from all three categories introduced below, resulting in a benchmark set of 100 QACs.

The calculations were performed using the same protocols as above, simply replacing the functional and the basis set of the B97-3c-method by the ωB97M-V functional and the def2-TZVPPD basis. After geometry optimizations of the structures obtained from the conformational search as described above, all thermodynamic properties, solvation energies, redox potentials and reorganization energies were obtained this way.

The results of these calculations are presented in the Supporting Information. There, the redox potentials are compared directly showing that while Δ*G*_RP_ remains unchanged on average ((0.06 ± 0.07) eV), the oxidation potentials are slightly larger than the ones obtained with the B97=3c-method ((0.47 ± 0.26) eV), cf. Figure S4. Regarding the correlations between the redox potentials and the frontier orbitals no changes of statistical significance are observed, cf. Figure S5. The reorganization energies show the same trend as the oxidation potentials, i.e. the ones computed with the ωB97M-V functional are a little bit larger than the ones obtained using the B97-3c-method ((0.21 ± 0.21) eV), cf. Figure S6.

The most important finding is that there are some small quantitative differences in the results, as expected. However, none of the results obtained using the B97-3c-method that we will discuss in the following are altered. Therefore, these high-quality calculations fully confirm our findings for a representative subset of our data set. Furthermore, our calculations show that the B97-3c-method yields reliable results for quantities of utmost importance in the field of electrochemistry of QACs.

## 3 Results and Discussion

### 3.1 Characterization of QACs

As mentioned in the Introduction it is not entirely clear whether or not the cations of the SE are involved in the electron transfer process responsible for a cathodic reduction. Though usually it is assumed that the SE can be viewed as inert, this cannot be taken for granted in general. In particular, if the Faradaic efficiency, i.e. the actual yield relative to the expected yield, is lower than anticipated, one possible side product might result from a redox reaction of the SE.

In order to investigate a possible contribution of the QACs in a reduction reaction, in a first step we characterized the structure of the reduced species of our entire set of QACs.

We observed three different kinds of behavior, cf. Figure 2. As expected, for the tetraalkyl-ammonium cations and other single-bonded systems (3014 (2082 tetraalkyl) singly charged cations and 464 (365 exhibiting alkyl chains) doubly charged cations in our data set) a fragmentation was observed (Figure 2a). For those QACs that contain the quaternary nitrogen as a member of a ring (876 singly charged and 115 doubly charged cations) the reduction results in a ring-opening (Figure 2b). Finally, the third category includes QACs that have double-bonds or multiple-bonds (858 singly charged and 65 doubly charged cations) for which the reduction results in only minor changes in the molecular geometry (Figure 2c). In the following discussions, we will always distinguish between the three categories found here, fragmentizing (3478 cations), ring opening (991), multiple bond (923).

**Figure 2:**
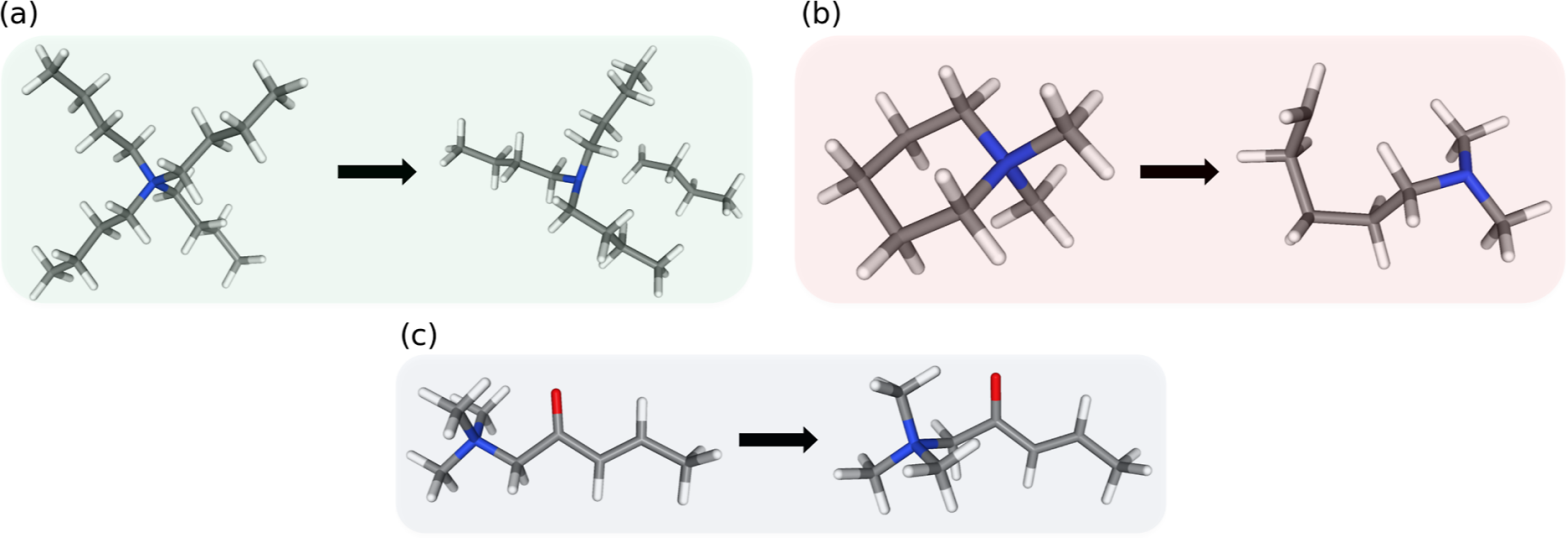
Reduction of QACs belonging to the different categories discussed in the text: (a) tetrabutylammonium, (b) mepiquat, (c) trimethyl­(2-oxopent-3-enyl)­ammonium. The color-code will be used throughout.

### 3.2 Redox Potentials

The reduction potential (RP) of a substance gives a hint toward its electrochemical stability against reduction. Therefore, it is instructive to consider the values of the RPs of the QACs and compare them to those of reactants in a cathodic reduction. In addition to the experimental results obtained for the electrochemical hydrodimerization of acrylonitrile in ref 20 (1), we chose the electrochemical synthesis of optically pure menthylamines^59^ (2), and the deoxygenation of an aromatic amide, *N*-benzoyl-2-ethylhexylamine^60^ (3) from the literature. In Figure 3a, we present the results of the computation of the RPs for the addition of one electron for all QACs of our data set together with the RPs of acrylonitrile (1), (−)-menthone oxime (abbreviated as “oxime”) (2), and *N*-benzoyl-2-ethylhexylamine (abbreviated as “amide”) (3) in MeCN. In ref 20 we presented the RPs relative to a standard hydrogen electrode^36^ for all QACs among other molecular, electric and electronic properties that we discussed in that work. Here, we distinguish the RPs relative to the ferrocenium/ferrocene redox couple according to the reduction products of the cations and it is apparent that the distributions of the RPs exhibit quite similar shapes for all three categories of QACs. The values obtained by our methodology are within the range of what has been found earlier for QACs.^12,61^ It is evident from Figure 3 that a large number of QACs can be reduced even easier than the substrates of the reactions considered. Therefore, from a purely thermodynamic point of view and neglecting all other aspects of practical relevance it might well be that in some cases the electron transfer reaction involved in the reduction process cannot be viewed as purely heterogeneous and the SE has to be chosen carefully. As mentioned above, Mousavi et al.^15^ thoroughly investigated the dependence of the RPs of a set of 16 QACs on various structural parameters such as the volume of the QACs and the chain length of tetraalkyl-QACs. Without presenting the results here, we note that our calculations confirm the absence of significant correlations between the RP and the quoted properties for the whole set of QACs studied.

**Figure 3:**
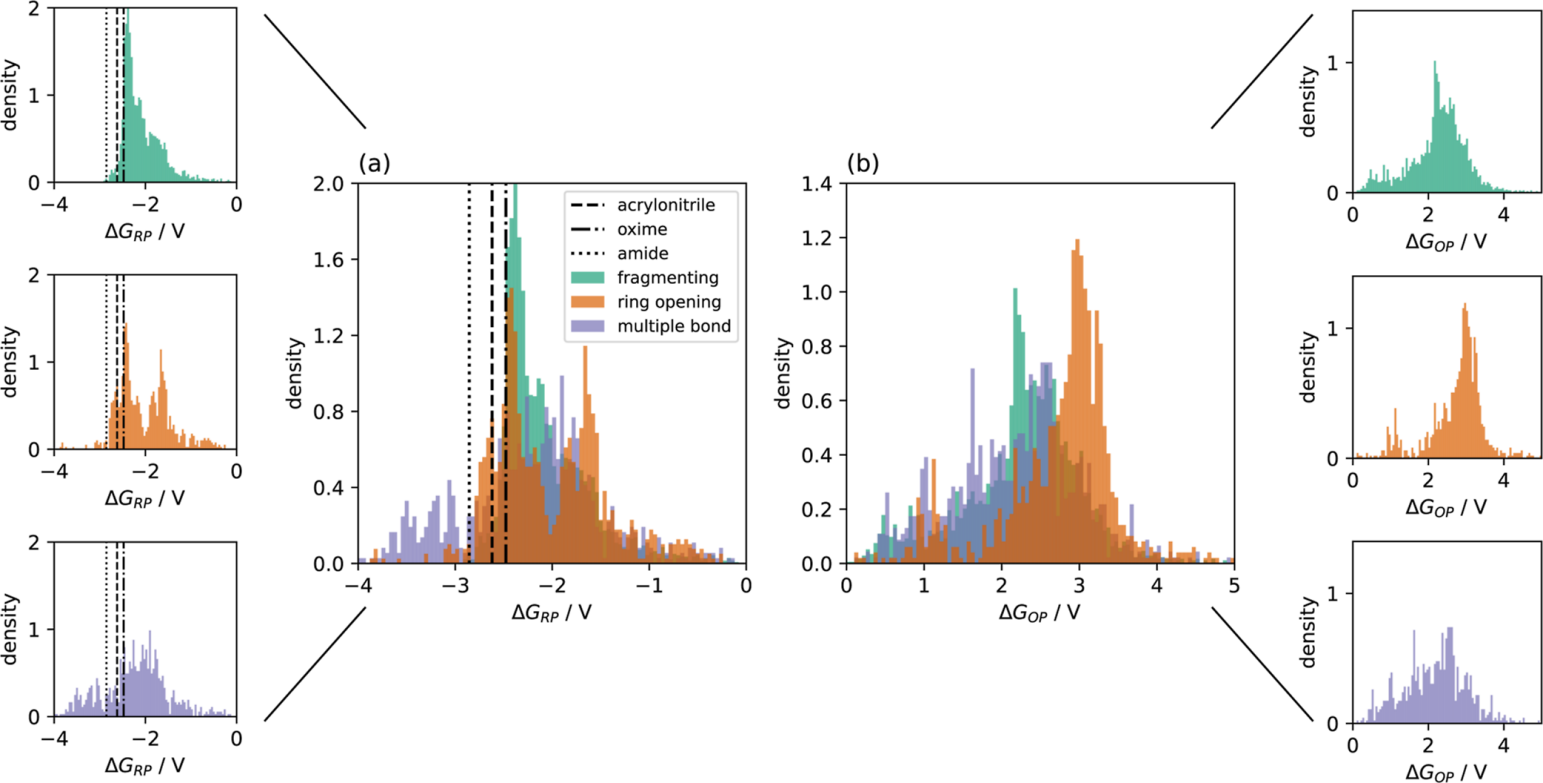
Distribution of the reduction potentials (a) and the oxidation potentials (b) for all QACs of the present data set (5392 singly charged and double charged cations). Figure adapted from ref 20 (Figure 4). The horizontal lines are the reduction potentials for the educts of reactions (1) (acrylonitrile), (2) (oxime), and (3) (amide). The small panels show the individual distributions for the three categories.

In order to be able to discuss the electrochemical stability of the QACs further, in Figure 3b we present the oxidation potential (OP) for the QACs based on the reaction *Q* ^+^ → *Q* ^2+^ + e^–^. Here, *Q* ^+^ denotes a singly charged or a doubly charged ion. It is interesting to note that there are only minor differences between the different categories of QACs in the overall behavior of the distributions of the RP. In case of the OP, the systems containing the quaternary nitrogen as a member of a ring appear somewhat more stable than the other ones. The electrochemical window can be defined as the difference between Δ*G*_OP_ and Δ*G*_RP_ ^62,63^ and is presented for all QACs in Figure 4. Here, in accord with the observation regarding the OP, some differences between those systems which undergo a ring-opening when reduced to the other two categories become apparent in the sense that a larger number of these systems appears to show a somewhat larger electrochemical stability.

**Figure 4:**
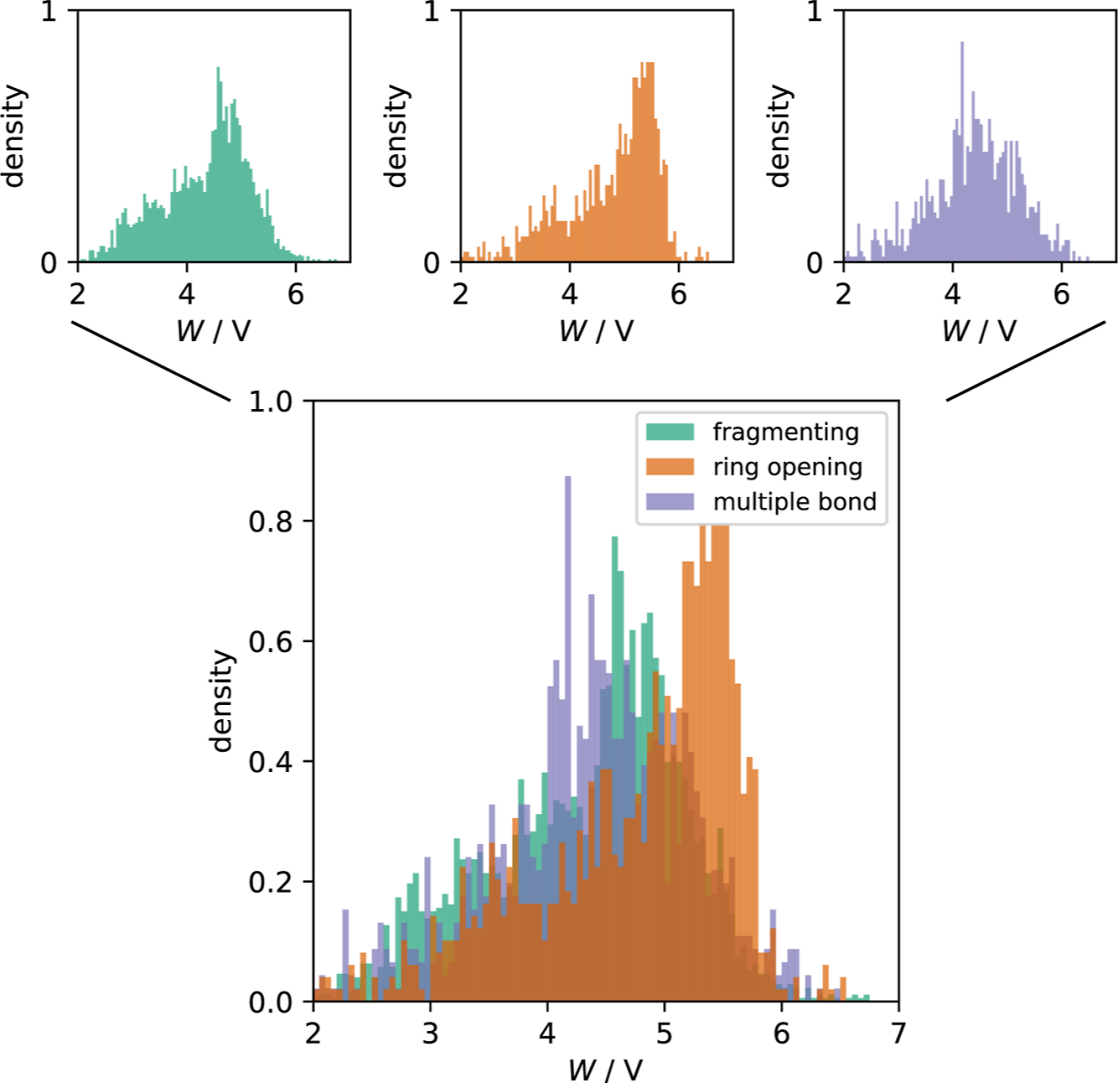
Electrochemical window defined as the difference between the OP and the RP, *W* = Δ*G*_OP_ – Δ*G*_RP_, for the whole set of QACs studied. The small panels show the individual distributions for the three categories.

There have been a number of studies that investigated the relation between the redox potentials and the frontier molecular orbitals, i.e. between the OP and the highest occupied molecular orbital (HOMO) and similarly between the RP and the lowest unoccupied molecular orbital (LUMO). The existence of a relation between these quantities would simplify the calculation of redox potentials substantially. The theoretical basis for a possible existence of a connection between the energies of the molecular orbitals and the redox potentials is given by the extension of Koopmans’ theorem to DFT.^64–66^ Of course, it has to be assumed additionally that the vertical ionization potential approximately coincides with the adiabatic one and similarly for the electron affinity. For some systems, a linear relation has been observed between the redox potentials and the frontier orbital energies obtained from DFT calculations^67–69^ and in some investigations such a relation has been observed between the RP and the adiabatic electron affinities (EAs).^15,68^ In Figure 5, we plot the RP as a function of the LUMO energy and the OP as a function of the HOMO energy for all QACs of our data set. We find a weak correlation between the RPs and the LUMO energies and a somewhat stronger one between the OPs and the HOMO energies. The first observation is in accord with the well-known fact that LUMO energies computed with Kohn–Sham DFT neither give reliable results for the EA^70^ nor for the RP.^15^ For the occupied Kohn–Sham orbitals, however, our finding of an approximate linear correlation between the HOMO energies and the OP appears reasonable.^70^

**Figure 5:**
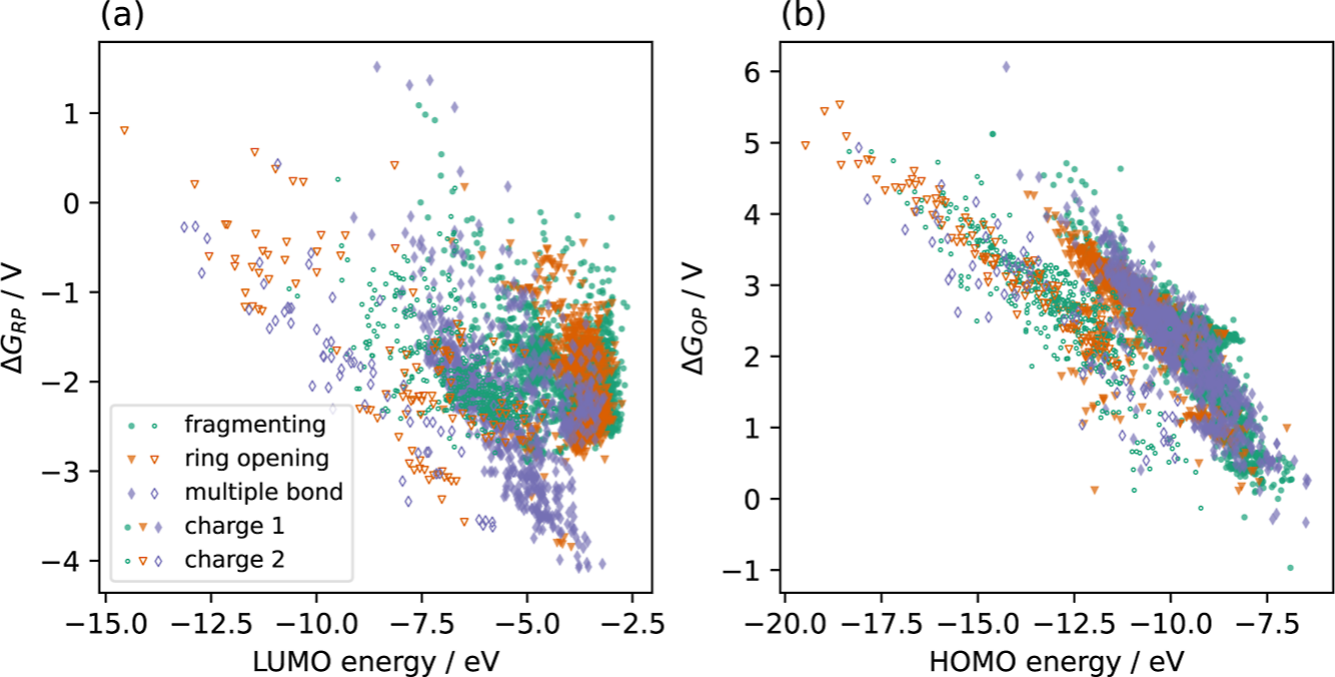
Reduction potential versus LUMO energy (a) and oxidation potential versus HOMO energy (b) for the whole set of QACs studied.

The actual calculation of the redox potentials requires the computation of the adiabatic EAs and IPs instead of the vertical ones along with the corresponding solvation energies, cf. eq 1 discussed above. Therefore, in Figure 6, we plot the RPs versus the adiabatic EAs (a) and the OPs versus the IPs (c). In both cases we observe a strong correlation with different slopes for singly charged and doubly charged systems, indicating the importance of electronic relaxation effects that are neglected in Koopmans’ theorem. In case of the RPs, the slopes determined by linear regressions for singly charged QACs are very similar to what has been observed for other systems earlier.^36^ For doubly charged systems, the slope is smaller, which already indicates the larger impact of the solvation energy difference, Δ*G*_x_^solv^ (x = ox, red), in this case. The RPs are plotted as a function of the Δ*G*_red_^solv^ in Figure 6b showing that indeed there is some correlation between the two quantities in case of doubly charged QACs. We attribute this finding to the stronger interaction between the solvent and the higher charged QACs.

**Figure 6:**
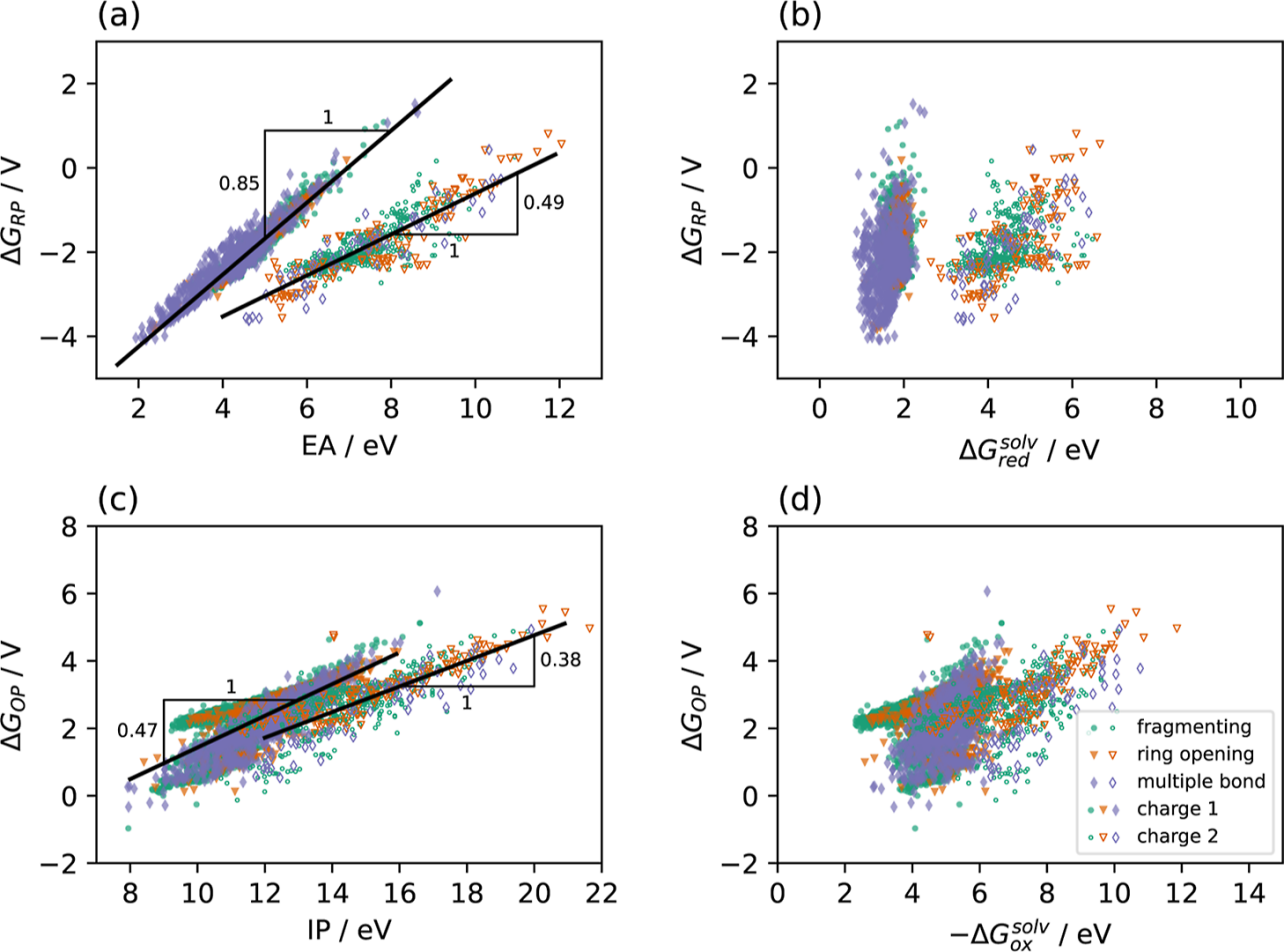
Reduction potential versus the adiabatic electron affinity (a) and versus the difference in solvation energies of the cations and the reduced species (b) and oxidation potential versus ionization potential (c) and versus solvation energy difference (d) in MeCN for the whole set of QACs studied. The lines are linear regressions: (a): singly charged (full symbols): RP = −5.95 + 0.85 × EA (*R* ^2^ = 0.907), doubly charged (open symbols): RP = −5.47 + 0.49 × EA (*R* ^2^ = 0.749), and (c): singly charged: OP = −3.27 + 0.47 × IP (*R* ^2^ = 0.607), doubly charged: OP = −2.84 + 0.38 × IP (*R* ^2^ = 0.670).

In case of the OPs, the slope of the OP versus IP relation (Figure 6c) is smaller than those for the RP as a function of the EA and the correlation to the solvation energy differences Δ*G*_ox_^solv^ (Figure 6d) is stronger. This finding is in accord with the stronger dependence of the RPs on Δ*G*_ox_^solv^ in case of doubly charged ions, since in the oxidation considered here, all ions have higher charges. Of course, the impact of the solvation energy on the redox potentials depends on the nature of the solvent. One might expect a stronger correlation for more protic solvents where electrostatic interactions play a more prominent role.

Our results indicate that usually it is not sufficient to perform ground-state DFT calculations of only one species of isolated molecules in order to gain information about the redox potentials and both, the geometries of the relevant species and the corresponding solvation energies are important.

### 3.3 Reorganization Energies of QACs

The redox potentials give reliable information about the thermodynamic stability of the QACs under consideration. The kinetics of electrochemical reactions, however, is usually determined by thermally activated electron transfer processes.^42,43^ The reorganization energy is an important factor defining the activation free energy of heterogeneous electron transfer. As detailed in Section 2, the total reorganization energy is given by the sum of two contributions, cf. eq 2. The inner-sphere reorganization energy, eq 4 and 5 and Figure 1, is associated with the energetic changes in molecular conformations while the outer-sphere reorganization energy, eq 3, has its origin in the electrodynamical response of the solvent.

As the QACs are spatially located in the double layer and because it is well-known that the outer-sphere reorganization energy becomes very small in the vicinity of the electrode,^51,71,72^ we neglected λ_out_ and only computed the inner-sphere reorganization energy λ_*i*_ for the whole data set of QACs.

In the calculation of the reorganization energies of the substrates of the reactions (1), (2), and (3), on the other hand, we used eq 2 and 3 for the calculation of λ because the corresponding molecules are located at a larger distance to the electrode. The molecular radius *r*_mol_ was estimated from the volume of the cavity computed by the COSMO method^73,74^ as the radius of a sphere with the same volume.

As there does not appear to be a clear-cut definition of the thickness of the double layer,^75^ we will use distances *R* ≥ 15 Å because in one study it was found that the reorganization energy approaches its bulk value at a distance of about 20 Å from the electrode.^72^

In Table 1, we give the values for λ_*i*_ and the bulk value for λ_out_ (*R* = 50 Å). It is obvious that both contributions are important and that λ_out_ can by no means be neglected.

**Table 1:** Inner-Sphere and Outer-Sphere Reorganization Energies of Reactants of the Example Reactions

	λ_*i*_/eV	λ_out_/eV
acrylonitrile (1)	0.27	1.39
oxime (2)	2.40	0.96
amide (3)	0.38	0.86

For those QACs for which reduction results in a ring-opening or for multibond systems an important contribution to the inner-sphere reorganization energy stems from the possible change in the molecular geometry. However, for those QACs that fragmentize into a tertiary amine and a radical the situation is different. Here, the complex consisting of the amine and the radical can only be viewed as one entity if the two subsystems are not separated significantly. As explained in Section 2, we performed MD simulations at room temperature (298 K) and ambient pressure (1 bar) in order to resolve this issue. In our examples, we found that on the relevant time scale of vibrational relaxation the amine and the radical can be viewed as one system.^32^ Thus, we assume that to a good approximation we are allowed to compute the reorganization energy using the geometry of the total system for the reduced species.

The results of our calculations are presented in Figure 7. From this figure it is evident, that λ_*i*_ is very similar for the QACs that undergo a ring-opening and those that fragmentize upon reduction. However, the λ_*i*_ values for the multibonded systems can be up to more than 2 eV smaller. This can be understood from the fact that the molecular reorganization in the latter systems is accompanied with only slight changes in the geometry. The bars in Figure 7 represent the total reorganization energies for the reactions (1), (2), and (3) for distances to the electrode in the range from 15 Å to 50 Å.

**Figure 7:**
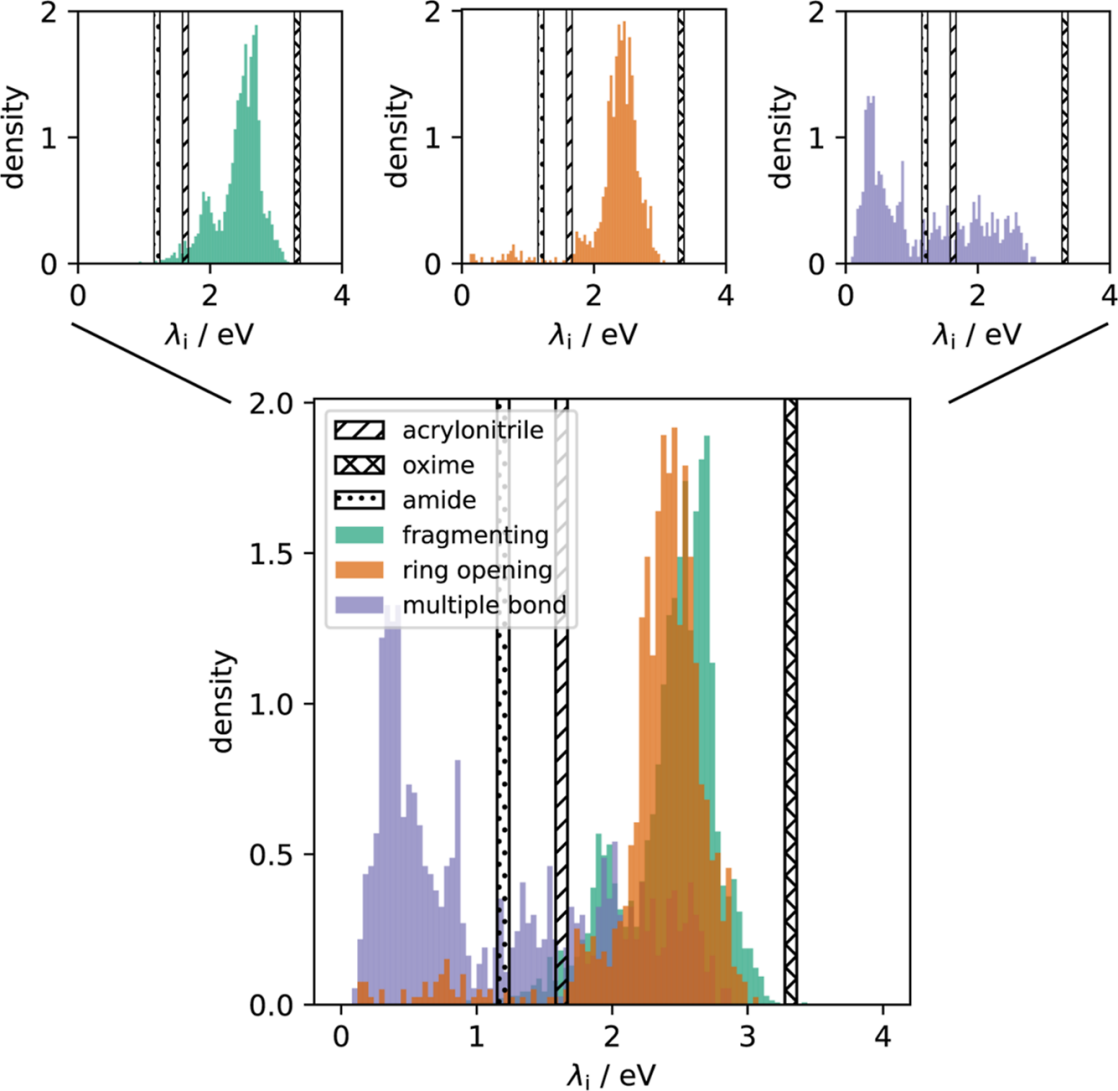
Inner-sphere reorganization energies according to eq 4 of all QACs of our data set. Horizontal bars represent the total reorganization energies λ, eq 2, computed for the reactants of reactions (1) (acrylonitrile), (2) (oxime), and (3) (amide), for distances from the electrode between 15 Å and 50 Å. The small panels show the individual distributions for the three categories.

The inner-sphere reorganization energy plays a dominant role in theoretical treatments of electron transfer^76,77^ and also in various further problems related to the intra- and intermolecular transfer of energy or charge.^78^ In the framework of models for linear electron-phonen coupling, one has a relation between λ_*i*_ and the coupled phonon density of states.^50,78^ If one assumes a common coupling constant for all vibrational modes, one can express λ_*i*_ in terms of a so-called Huang–Rhys factor *S* determining the electron–phonon coupling strength.^50,79^ In this case, one finds

6 $$\lambda_{i}^{x}≃S^{x}\cdot \hbar \omega_{eff}^{x}\;with\;\omega_{eff}^{x}=\sum_{\alpha }\omega_{\alpha }^{x}\;\mathrm{for}\;x=ox,red$$

where ω_α_^x^ denote the harmonic frequencies of the respective system.^79^ The corresponding results are presented in Figure 8. We find that the Huang–Rhys factors determined this way are distributed in a narrow range and numerically are on the same order of magnitude of what has also been reported earlier for other molecular systems.^79,80^ The similarity of the Huang–Rhys factors in the oxidized and the reduced state of the QACs are in agreement with the finding of ω_eff_^ox^ ≃ ω_eff_^red^, cf. Figure 8b. This is interesting to note, in particular in view of the fact that only for the multibonded QACs the nuclear configurations of the two states are very similar, giving rise to the observed prevalence for small values for the reorganization energies. Apparently, the changes in the molecular geometries in case of the fragmenting and the ring-opening categories mainly affect low-frequency modes with relatively small weight in the sum in eq 6.

**Figure 8:**
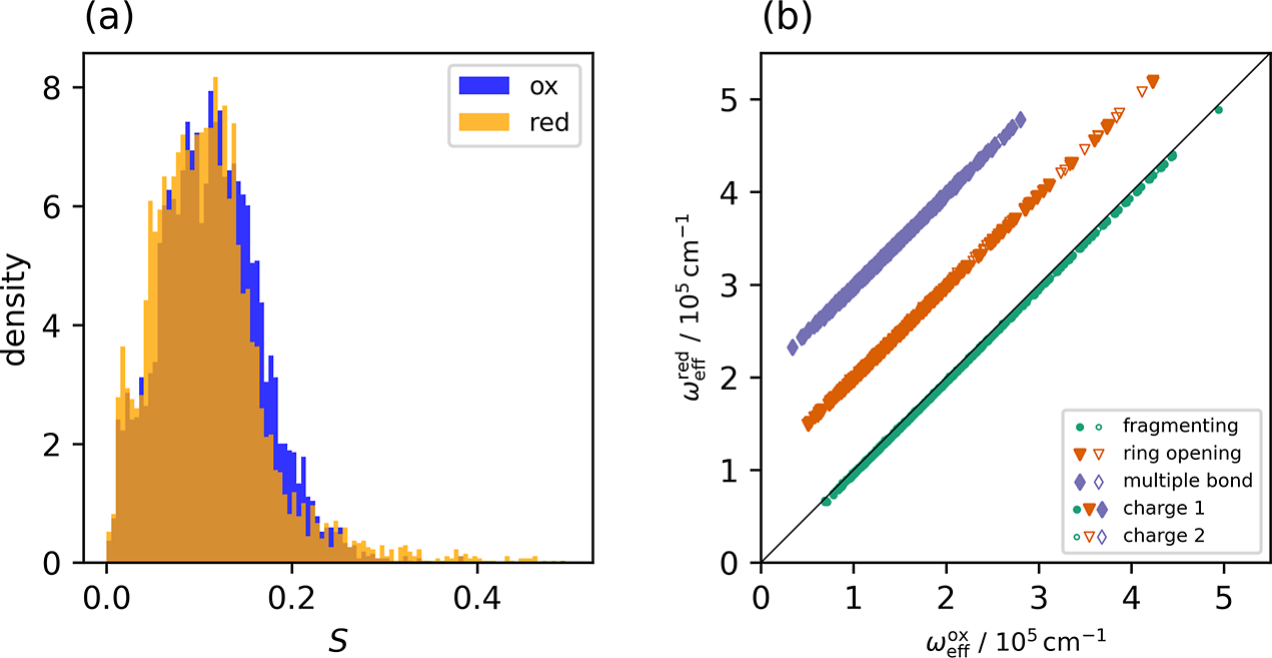
(a) Distribution of the Huang–Rhys factors *S* ^ox^ (blue) and *S* ^red^ (orange) for all QACs determined according to eq 6. (b) Effective frequencies of the reduced species plotted against the one of the oxidized species. The values for ω_eff_^red^ for systems undergoing ring-opening are shifted by a factor of 10^5^ cm^−1^ and those for multibond systems by 2 × 10^5^ cm^−1^. The thin line has a unit slope.

The applicability of eq 6 shows that the approximations inherent in the Marcus treatment of heterogeneous electron transfer appear meaningful. In particular, for the important class of QACs a harmonic approximation of the potential energy surfaces with a curvature given by the respective reorganization energies seems to be sufficient in order to describe important features of the electron transfer process.

From our observation of small values for the reorganization energy for a number of QACs exhibiting multiple bonds, we conclude that such systems should not be considered to be the first choice when the reduction of the SE should be suppressed. Of course, these arguments neglect the presumably quite different electronic couplings of the different species. This, however, mainly affects the prefactor of the electron transfer rate and we consider the activation energy to be the most important factor.

### 3.4 Experimental Determination of the Reduction of Representative QACs

Our calculations show that those QACs which undergo ring-opening are electrochemically more stable in a statistical sense than the other two categories. The finding that acyclic tetraalkylammonium are less stable than the former is in accord with the typical behavior observed in certain ionic liquids.^14,61^ The QACs with multiple bonds exhibit small reorganization energies and therefore are expected to show limited kinetic stability. Additionally, from a chemical point of view, these systems usually will not routinely be used as SEs due to their chemical (thermodynamic) instability in solvents exhibiting nucleophilic and acidic nature.^81^ Therefore, in the following, we will mainly study systems belonging to the other two categories. Since the uptake of an electron by cathodic reduction can happen also with the QACs considered in the present investigation and in electro-organic synthesis this constitutes an unwanted side reaction, it is necessary to investigate this point carefully.

To further validate the results of our calculations, we supplement them by an experimental study of the electrochemical stability against cathodic reduction of six QACs. We used experimental conditions that are typical for electro-organic synthesis. The stability of the QACs was investigated using two analytical methods: proton nuclear magnetic resonance (^1^H NMR) spectroscopy and gas chromatography (GC). Using ^1^H NMR spectroscopy, the catholytes containing three different supporting electrolytes, namely methyltributylammonium methylsulfate (MTBS), 1,1-dimethyl-pyrrolidinium methylsulfate, and 5-azoniaspiro[4.4]­nonane chloride, were investigated. GC was used to study the stability of four supporting electrolytes: benzyltriethylammonium chloride, MTBS, tetrabutylammonium tetrafluoroborate, 1-butyl-1-methyl-pyrrolidinium bis­(trifluormethylsulfonyl)­imide, cf. Figure 9. In our initial screening by GC, we observed a small decomposition of quaternary ammonium salts (about 2%), which is too close to the detection limit of ^1^H NMR spectroscopy. This led us to conclusion that a more precise method of analysis was required, and we therefore continued our research using GC as the analytical method. All experiments were performed in a divided screening-type cell (Nafion was used as separator material). The quaternary ammonium salt (0.6–4.0 mmol) was added into each compartment, followed by an addition of the solvent (5 or 7 mL) and H_2_SO_4_. Graphite, dimensionally stable IrO_2_-based anode (DSA), or platinum was used as anode material and boron-doped diamond (BDD), nickel foam, graphite, glassy carbon, copper, or lead was used as cathode material. The amount of applied charge was ranging from 224 to 1544 Coulomb (3–8 *F* relative to the electrolyte), and the applied current density was between 9 and 29 mA/cm^2^. All reactions were performed under air and at room temperature. For more information regarding the experimental setup we refer to the Supporting Information.

**Figure 9:**
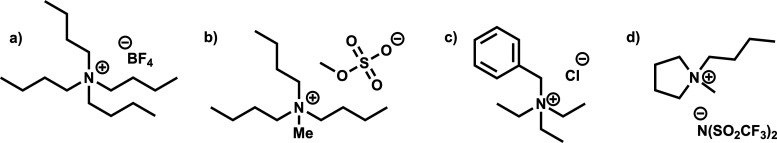
(a) Tetrabutylammonium tetrafluoroborate (b) methyltributylammonium methylsulfate (MTBS) (c) benzyltriethylammonium chloride (d) 1-butyl-1-methyl-pyrrolidinium-bis­(trifluormethylsulfonyl)­imide.

For the workup process in which we have isolated the decomposition product, we took advantage of the fact that the free amine compounds and the ionic supporting electrolytes have different solubilities in water and organic solvents depending on the pH. After the reaction, the first step in the workup procedure was to remove the water-miscible acetonitrile, which could potentially interfere with the subsequent extraction using another water-immiscible organic solvent. To keep the crude amine in the mixture during the removal of acetonitrile, the catholyte was acidified, forming an amine salt with a higher boiling point than the free amine. Although we start with acidic reaction conditions, the additional acidification step was necessary in the workup procedure due to the different pH values of the analyzed catholytes after the reactions. After removal of the acetonitrile under reduced pressure, the pH of the aqueous residue was increased to recover back the free amine. The amine was extracted from the crude mixture with diethyl ether. This step was required in order to separate a supporting electrolyte and a free amine before the quantification analysis. The analysis was performed by GC-FID using decane as an internal standard. Prior to each reaction, the starting materials were subjected to the same procedure as described above in order to determine the purity of the compounds. The results show different degrees of chemical decomposition into the corresponding free amines for different supporting electrolytes. Only for the benzyltriethylammonium chloride (Figure 9c) we obtained a significant yield of triethylamine (up to 56% yield, Table S1). As expected, the benzyl group was the most sensitive group under reduction conditions, and thus only triethylamine was observed, without any *N*,*N*-diethylbenzylamine. Other acyclic tetraalkylammonium salts, namely tetrabutylammonium tetrafluoroborate (Figure 9a), and methyltributylammonium methylsulfate (MTBS, Figure 9b) showed increased stability under reductive conditions. The reaction conditions for the decomposition of benzyltriethylammonium chloride (Figure 9c) were also applied to MTBS (Figure 9b), however, the results show a high stability of MTBS under these conditions (Table S2). In contrast to benzyltriethylammonium chloride (Figure 9c), we found that the breakdown of MTBS (Figure 9b) to *N*,*N*-dibutylmethylamine proceeded slightly better without acid and with the addition of water. We observed fragmentation of MTBS to *N*,*N*-dibutylmethylamine in up to 6% yield, depending on various parameters, including electrode material, current density, concentration, and temperature (Table S3). The tetrabutylammonium tetrafluoroborate showed similar reactivity to MTBS (Table S4). In the case of the cyclic 1-butyl-1-methyl-pyrrolidinium bis­(trifluormethylsulfonyl)­imide (Figure 9d), we did not detect any of the corresponding cleavage products (Table S5).

Apart from the first example, these results strongly hint toward the high stability of the systems investigated. We can discriminate among the ring system and the acyclic tetraalkyl-ammonium, which we find both to be stable in agreement with our computational study.

## 4 Conclusions

In the present study, we used modern quantum chemical methodologies to analyze the electrochemical properties of a large number of QACs, a class of compounds that is often utilized as SE in electrochemical synthesis. In particular, we focused on the redox potentials and the reorganization energies, as these quantities are important for the thermodynamic and the kinetic aspects of the relevant electron transfer processes. The set of QACs consists of more than 5000 different singly charged and doubly charged molecules which can be classified according to their behavior under reduction. We find three different categories, the very important category of systems that fragmentize into a tertiary amine and a radical, one category which contain the nitrogen atom as a member of a ring that opens upon reduction, and systems containing multiple bonds that form radicals.

The distributions of both, the RPs and also the OPs show a peaked structure for all categories. In case of the RPs, the distributions are very similar but for the OPs the QACs undergoing a ring-opening under reduction show a maximum at a somewhat higher value than the other categories and this fact is also reflected in the prevalence of a larger electrochemical window. When considering electrochemical reduction reactions, we find that a large number of QACs can be reduced even easier than the reactants of three example reactions taken from the literature. Therefore, from a purely thermodynamical point of view, care has to be taken in choosing the right SE for such reduction reactions.

We found only weak correlations between the frontier orbitals calculated using DFT and the redox potentials with the one between the OP and the HOMO being somewhat stronger. However, a significant linear correlation was found between the RP and the EA and the OP and the IP, when the adiabatic properties are considered instead of the vertical ones. This correlation is expected to depend on the polarity of the solvent because in addition to the EA or IP the solvation free energies of the QAC in the solvent considered are needed in the computation of the redox potentials. From these calculations we conclude that for the class of QACs considered here, it is not sufficient to just perform DFT calculations and use the frontier orbitals as a measure for the redox potentials. Additionally, in accord with earlier investigations on QACs,^15^ we do not observe correlations between the redox potentials and structural features of the QACs like chain lengths or molecular volume.

We calculated the inner-sphere reorganization energy of the QACs because it is a well-established fact that the outer-sphere reorganization energy vanishes in the vicinity of the electrode where the cations of the SE are mainly located. For comparison, we computed the total reorganization energies for the reactants of example reactions using a simple electrostatic model for the outer-sphere reorganization energy and found that in general both contributions to the reorganization energy, outer-sphere and inner-sphere, contribute significantly. Our calculations indicate that QACs containing multiple bonds might not be useful candidates for the use as SEs in electrochemical reduction synthesis because they show a clear prevalence for very small inner-sphere reorganization energies. Thus, in a number of situations these systems might be reduced more easily than the reactants of the reaction considered.

We find that a simple “one-mode model”,^79^ i.e. a description in terms of a single Huang–Rhys factor and one effective vibration frequency, defined as the sum of all vibrations, works astonishingly well. This means that even in systems undergoing rather substantial conformational changes the frequencies associated with the different geometries only play a minor role in the determination of the inner-sphere reorganization energies.

Our calculations do not allow to discriminate between the systems undergoing ring-opening and those that fragmentize upon reduction. Therefore, we performed experiments on examples of members of both groups under identical conditions and we found that with the chosen setup it was possible to reduce acyclic tetraalkylammonium QACs but not an QAC which contain the quaternary nitrogen atom as a member of a ring. Systems containing multiple bonds usually will not be employed as SEs due to their chemical instability under acidic conditions. We therefore conclude from our investigations that QACs containing the quaternary nitrogen atom as ring member are expected to be the most relevant for use as SE in electrochemical cathodic reduction reactions.

Our investigations show that modern quantum chemical methodologies allow to compute important thermodynamic and kinetic parameters for large numbers of relevant QACs that are possible candidates for use as SEs in electrochemical synthesis. Using our results and complementing them by the calculation of the electronic coupling between the electrode and the cations will allow to determine electron transfer rates from first principles. The rates obtained this way can then be compared directly with the ones obtained for the reactants of reductions of chemical interest and will be helpful in the design of future experiments.

## Supplementary Material



## Notes

Experimental setup, experimental and spectroscopic results. Additionally, some computational details are presented (PDF)
