# Supplementary material for: Quaternary Ammonium Salts as Supporting Electrolytes in Cathodic Reductions: An Analysis of Their Electrochemical Stability
Source: J Phys Chem B. 2025 Jun 11;129(25):6241–52. doi: 10.1021/acs.jpcb.5c00650 (PMC12207581; doi:10.1021/acs.jpcb.5c00650)
Supplement: Supplementary file 1 [file jp5c00650_si_001.pdf]

Supporting Information

Quaternary Ammonium Salts as Supporting Electrolytes in Cathodic Reductions: An Analysis of their Electrochemical Stability

Florian Mast[a], Maximilian M. Hielscher[a], Eva Plut[b], Jürgen Gauss[a], Gregor Diezemann*[a], and Siegfried R. Waldvogel**[a,b]

[a] Department of Chemistry
Johannes Gutenberg University Mainz
Duesbergweg 10–14, 55128 Mainz, Germany

[b] Max-Planck-Institute for Chemical Energy Conversion
Stiftstrasse 34 - 36, 45470 Mülheim an der Ruhr, Germany

* E-mail: diezemann@uni-mainz.de
** E-mail: siegfried.waldvogel@cec.mpg.de

Content

1 Experimental Details S2
1.1 General Information S2
1.2 Electrochemical Cell Setup S3
1.3 NMR Studies on Supporting Electrolyte Stability S3
1.3.1 Methyltributylammonium methylsulfate S4
1.3.2 Pyrrolidinium methylsulfate S4
1.3.3 5-Azoniaspiro[4.4]nonane chloride S5
1.3.4 Discussion S6
1.4 GC Studies on Supporting Electrolyte Stability S6
2 Computational Details S11
2.1 Computing Hardware S11
2.2 Dataset Preparation S11
2.3 Feature Calculations S12
2.4 Benchmark Calculations S13
2.5 MD Simulation Details S13
3 Benchmark Results S14
4 Data and Materials Availability S16
5 References S16

1 Experimental Details

1.1 General Information

Reagents and solvents were purchased in analytical grades from commercial suppliers (Acros Organics, ABCR, Sigma-Aldrich, Fischer Chemicals, Carl Roth). If solvent was purified, standard methods were followed.[S1]

Water for the electrolysis reactions was purified to MilliQ® grade (17.5 – 19.3 MΩ) using an MilliQ-Academic system from Millipore SAS (Molsheim, France, catalogue number ZMQS50001). Deionized water was used to supply the device. The MilliQ® water for the electrolysis was transferred to a multiple rinsed Schott beaker and used on the same day.

Ethyl acetate was purchased in technical grade and purified by distillation prior to use.

$CDCl_3$ was purchased from Deutero GmbH, Germany (>99.8%), LOT 23668.

1,3,5-Trimethoxybenzene was purchased from Tokyo Chemical Industry, Belgium (TCI) (>98.0%), LOT GXICA CJ.

Gas chromatography coupled with flame ionization detector (GC-FID) was performed on a Shimadzu GC-2030 (Shimadzu, Japan) using a HP-5ms capillary column (Agilent Technologies, Inc., Santa Clara, United States; length: 30 m, inner diameter: 0.25 mm, film: 0.25 µm, carrier gas: hydrogen). Gas chromatography coupled with a mass spectrometer (GC-MS) was performed on a Shimadzu QP 2020 instrument. Temperature program: 35 °C for 8 min, to 150 °C with 10 °C/min for 12 min. Hydrogen gas (99.9%) was purchased from Nippon Gases Deutschland GmbH, Germany. GC glass vials (1.5 mL) and screw caps (PTFE) were purchased from MN (Macherey-Nagel, Düren, Germany).

Power supply: A HMP4040 device (Rhode&Schwarz, München, Germany) with a controllable DC output of 0–32 V (±1 mV) and 0–10 A (±1 mA) and a maximum power of 160 W per channel (4 channels in total) was used. All electrolysis reactions were carried out under galvanostatic conditions using a two-electrode set-up.

NMR Spectroscopy: 1H NMR was recorded at 25 °C by using a Bruker Avance III HD 400 (400 MHz) (5 mm BBFO-SmartProbe with z gradient and ATM, SampleXPress 60 sample changer, Analytische Messtechnik, Karlsruhe, Germany). Chemical shifts (δ) are reported in parts per million (ppm) relative to TMS as internal standard or traces of $CHCl_3$ in the corresponding deuterated solvent.

1.2 Electrochemical Cell Setup

The stability test for the supporting electrolytes were carried out in divided batch cells (volume per compartment 7 mL). This cell setup is commercially available available from IKA (IKA Werke GmbH & Co. KG, Staufen, Germany) as *Screening System Package (6 cells)*. The electrode material used is noted in the respective procedures.

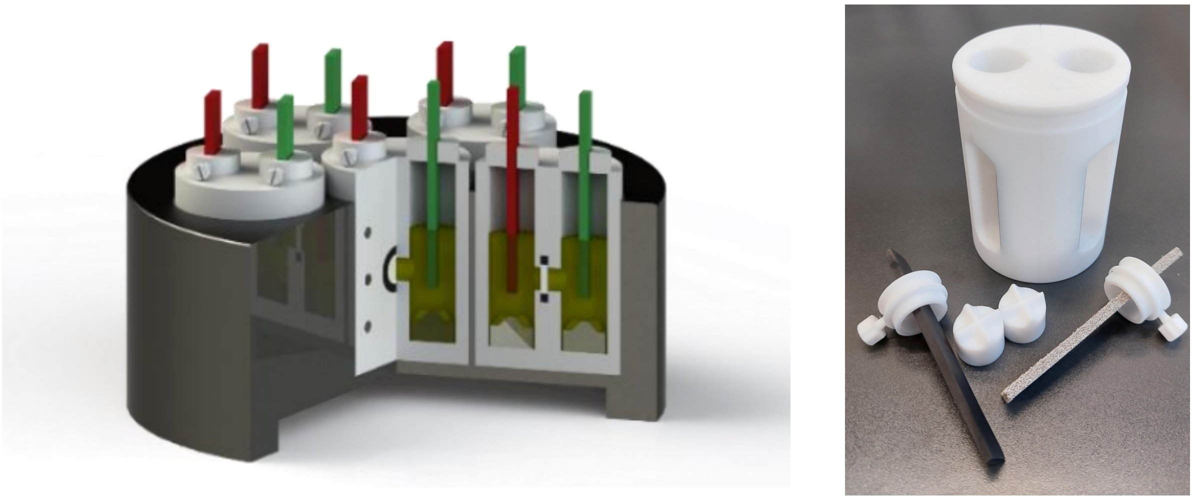

Figure S1: Left: Divided screening setup, taken from Ref.[S2]. Right: Batch-type divided, electrolytic cell with 2 x 7 ml internal volume. The electrode dimensions were 70x10x3 mm. The distance between anode and cathode during electrolysis was 0.7 cm. This cell type is commercially available from IKA as *Screening System Package (6 cells)*.

1.3 NMR Studies on Supporting Electrolyte Stability

General protocol:

For the preparation of the catholyte, the corresponding quaternary ammonium salt (1.1 mmol) was added to a snap cap vial and dissolved in 5mL of MeCN:H_2O (95:5). Then trifluoroacetic acid (2 mmol) was added. For the anolyte, 6 mL of a 1 M aqueous KOH was used to promote oxygen evolution reaction (OER). Both anolyte and catholyte were transferred to their respective compartments. Stainless steel (DIN/EN-1.4301; AISI-304) was used as anode material. Leaded bronze (CuSn10Pb10) or boron-doped diamond was used as cathode material. Leaded bronze and stainless steel were wet-polished with sandpaper (first P120 grit ISO/FEPA (100 grit ANSI), then P400 grit ISO/FEPA) before electrolysis and then rinsed with MilliQ® water and methanol and dried with a lint-free paper towel. The electrodes were immersed 3 cm deep into the solution (predetermined with the electrolyte unstirred), resulting in an active electrode area of 3 cm^2 per electrode. A charge of 250 C was applied at a galvanostatic current of I = 60 mA (current density of 20 mA/cm^2). The catholyte was investigated using 1H NMR.

1.3.1 Methyltributylammonium methylsulfate

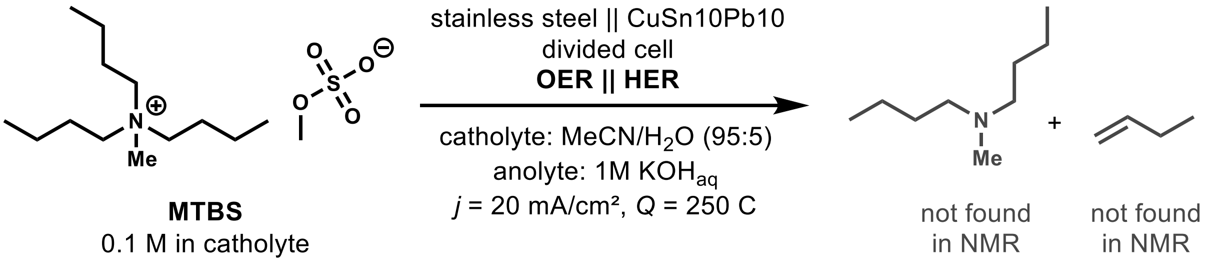

Scheme S1: Cathodic hydrogen evolution reaction (HER) with methyltributylammonium methylsulfate as supporting electrolyte. Anode reaction was OER in 1M KOH.

After the electrolysis, an aliquot of the catholyte was directly transferred to a coaxial NMR tube (with $CDCl_3$ in the inner tube) to check for the presence of the corresponding tertiary amine. To secure the assignment, a reference spectrum of the corresponding quaternary ammonium salt with the base was recorded. No signals of the tertiary amine were found.

1.3.2 Pyrrolidinium methylsulfate

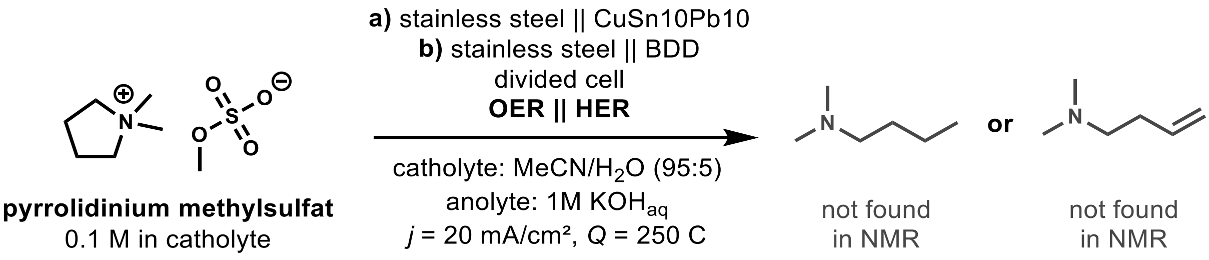

Scheme S2: Cathodic hydrogen evolution reaction (HER) with pyrrolidinium methylsulfate as supporting electrolyte. Anode reaction was OER in 1M KOH.

After the electrolysis using CuSn10Pb10 as cathode material, an aliquot of the catholyte was directly transferred to a coaxial NMR tube (with $CDCl_3$ in the inner part of the tube) to check for the presence of the corresponding tertiary amine. No additional signals indicating a degradation were found. An aliquot of the catholyte was transferred to a flask and the solvent was removed under reduced pressure (< 5 mbar, 50 °C). The solid was solved in CD_3CN and transferred to an NMR tube. Also in this sample, no tertiary amine was found. The experiment was repeated using BDD as cathode material. Also, in this experiment, beside residual solvent signals, no signals of the tertiary amine were observed.

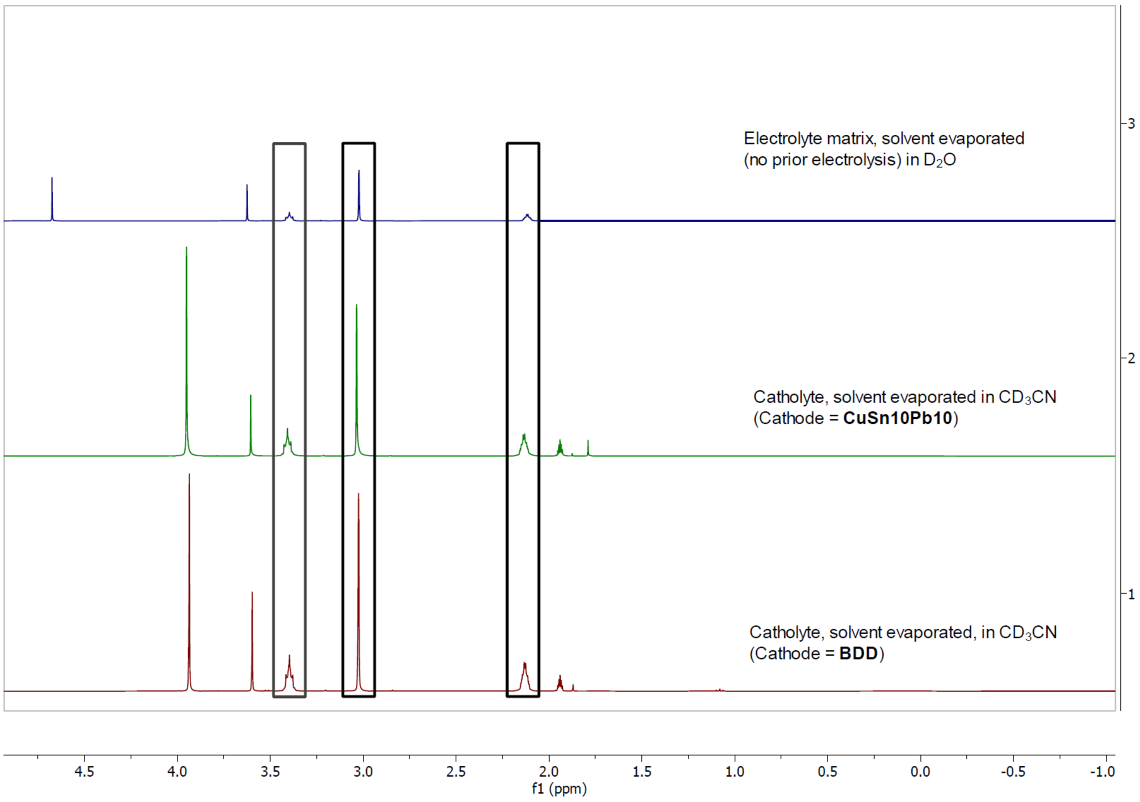

Figure S2: ^{1}H NMR spectra of the electrolyte matrix (upper), the catholyte after reaction using leaded bronze (middle) and BDD (lower).

1.3.3 5-Azoniaspiro[4.4]nonane chloride

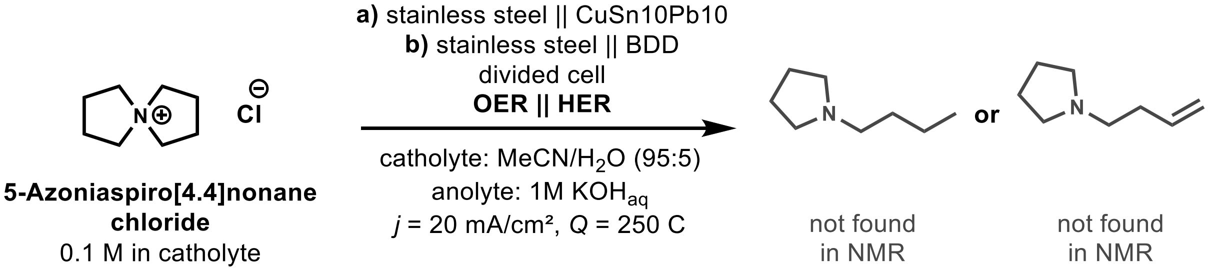

Scheme S3: Cathodic hydrogen evolution reaction (HER) with 5-Azoniaspiro[4.4]nonane chloride as supporting electrolyte. Anode reaction was OER in 1M KOH.

After the electrolysis using CuSn10Pb10 as cathode material, an aliquot of the catholyte was directly transferred to a coaxial NMR tube (with $CDCl_3$ in the inner part of the tube) to check for the presence of the corresponding tertiary amine. No additional signals indicating a degradation were found. An aliquot of the catholyte was transferred to a flask and the solvent was removed under reduced pressure (< 5 mbar, 50 °C). The solid was solved in CD_3CN and transferred to a NMR tube. Also in this sample, no tertiary amine was found. The experiment was repeated using BDD as cathode material. Also, in this experiment no signals of the tertiary amine were observed.

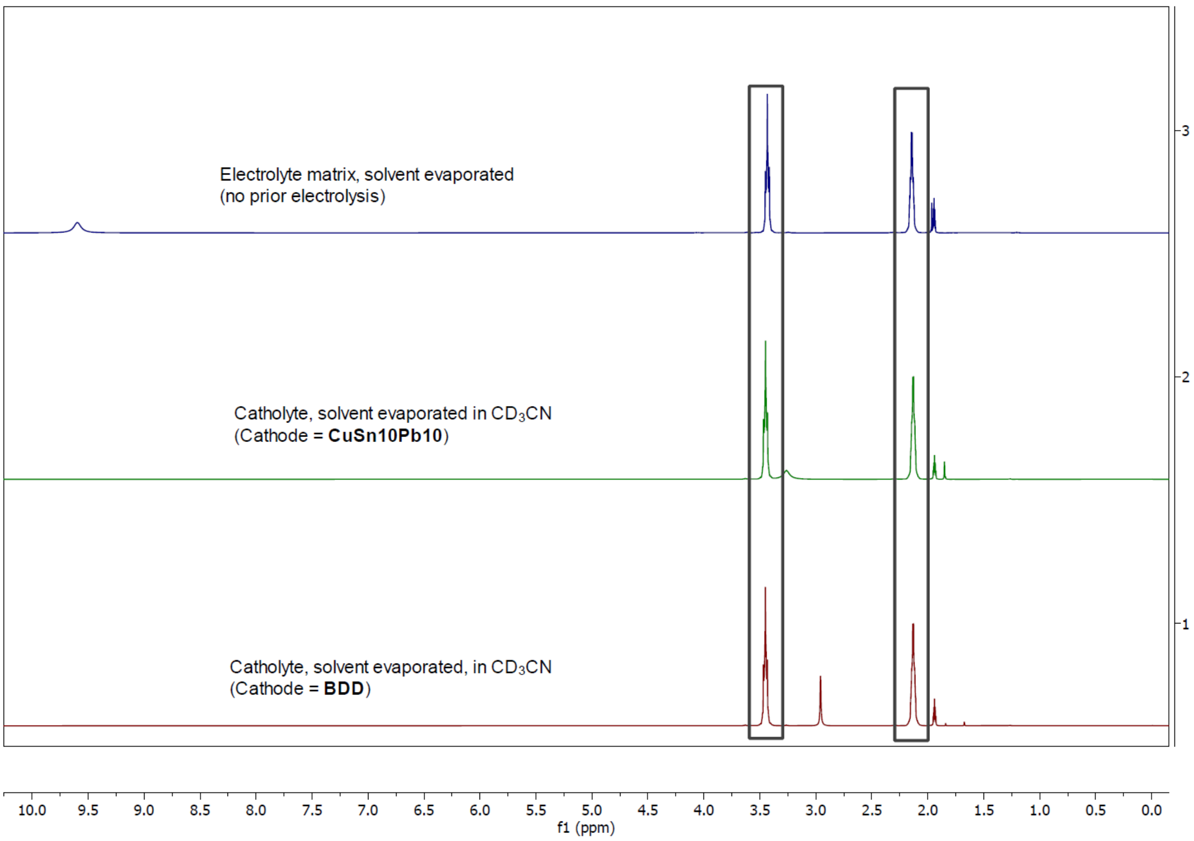

Figure S3: 1H NMR spectra of the electrolyte matrix (upper), the catholyte after reaction using leaded bronze (middle) and BDD (lower).

1.3.4 Discussion

No decomposition products of the supporting electrolytes were detected by ^{1}H NMR spectroscopy. These results were inconsistent with our initial screening by GC-FID, in which we observed a minor decomposition of quaternary ammonium salts (about 2%, see Table S3). As this value is below or too close to the detection limit of ^{1}H NMR spectroscopy, we, therefore, decided to examine the reaction solutions directly using more precise gas chromatography.

1.4 GC Studies on Supporting Electrolyte Stability

General protocol:

Reactions were set up in a divided screening-type cell with Nafion™ membrane (N324) as a separator. In a typical experiment, indicated quaternary ammonium salt (1.0 mmol) was added into each compartment, followed by an addition of the solvent (5 or 7 mL) and H_2SO_4. The solution was stirred well before the electrodes were inserted and the electrolysis was started.

For screening, graphite, dimensionally stable IrO_2-based anode (DSA), or platinum was used as anode material and boron-doped diamond (BDD), nickel foam, graphite, glassy carbon, copper, or

lead was used as cathode material. The amount of applied charge was ranging from 224 to 1544 Coulomb (3-8 *F* relative to the electrolyte), and the applied current density was between 9 and 29 mA/cm^2. Reactions were performed under air and at room temperature. Stirring rate was set to 300 rpm.

After the reaction, the catholyte was transferred into 100 mL round-bottom flask, and the cathode and the reaction compartment washed with 3 x 2.5 mL solvent mixture (MeCN/H_2O). Internal standard NPr_3 (15 µL) was added into the transferred reaction mixture, followed by the addition of H_2SO_4 until pH 2 was reached. The reaction mixture was concentrated under reduced pressure (150 mbar, 40 °C water bath). To the aqueous residue in the round-bottom flask, 1 M NaOH (aq.) was added until pH 12 was reached. The mixture was transferred in a 50 mL centrifuge tube, together with 7 mL H_2O and 2 x 5 mL Et_2O, which were used for washing the round-bottom flask. Decane (15 µL) was added as an internal standard and the mixture was well shaken. After fractionation, 1 mL of the organic layer was filtered through a small column filled with basic alumina (height = 2 cm, diameter = 0.5 cm) directly into a GC vial. Column was washed with Et_2O. The anolyte was quenched with the mixture of $Na_2S_2O_3$ (sat., aq.) and 1 M NaOH (aq.), without any analysis.

The analysis of catholyte was performed by GC. The compounds were identified by injecting the pure compounds in GC-FID, or by GC-MS. The quantification was done by GC-FID using decane as an internal standard and calculated, following the equation $\frac{S_x}{S_{IS}} = k\ \frac{n_x}{n_{IS}}$, where S_x = area of analyte, S_{IS} = area of internal standard, n_x = the mole of analyte, n_{IS} = the mole of internal standard. The values of k were obtained from the calibration curves for each product. NPr_3 served as an additional internal standard and verification of the workup procedure (the result can be trusted, if the ratio between NPr_3 and decane is as expected).

Purity control of the starting materials ($NBnEt_3Cl$, MTBS, NBu_4BF_4):

The starting material (1 mmol) was transferred into 100 mL round-bottom flask and dissolved in 12 mL solvent mixture. NPr_3 (15 µL) was added into the mixture, followed by the addition of H_2SO_4 until pH 2 was reached. The mixture was concentrated under reduced pressure (150 mbar, 40 °C water bath). To the aqueous residue in the round-bottom flask, 1 M NaOH (aq.) was added until pH 12 was reached. The mixture was transferred in a 50 mL centrifuge tube, together with 7 mL H_2O and 2 x 5 mL Et_2O, which were used for washing the round-bottom flask. Decane (15 µL) was added as an internal standard. After fractionation, 1 mL of the organic layer was filtered through a small column filled with basic alumina (height = 2 cm, diameter = 0.5 cm) directly into a GC vial. Column was washed with Et_2O. The analysis was performed by GC-FID as described above.

Table S1: Screening of electrochemical reaction conditions for the $NBnEt_3Cl$ decomposition.

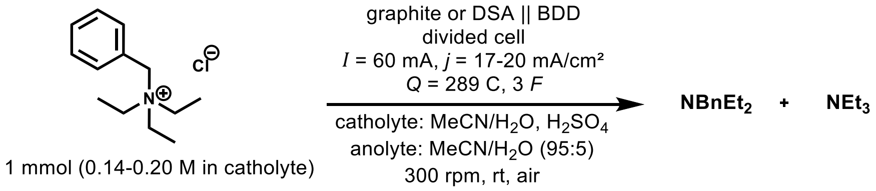

Entry	Anode	j (mA/cm²)	Solvent in catholyte	c (M)	$n_{H_2SO_4}$ (mmol)	$c_{H_2SO_4}$ (M)	GC yield of NEt_3 (%)
**1**	Graphite	17	MeCN	0.14	0.35	0.05	**56**
**2**	Graphite	17	MeCN	0.14	0.50	0.07	**52**
**3**	Graphite	17	MeCN	0.14	1.00	0.14	**5**
**4**	Graphite	17	MeCN	0.14	2.00	0.29	**0**
**5**	Graphite	20	MeCN	0.20	0.35	0.07	**47**
**6**	Graphite	20	MeCN	0.20	0.50	0.10	**13**
**7**	Graphite	20	MeCN	0.20	1.00	0.20	**0**
**8**	Graphite	17	$MeCN/H_2O$ (95:5)	0.14	-	-	**0**
**9**	Graphite	17	$MeCN/H_2O$ (95:5)	0.14	0.35	0.05	**0**
**10**	DSA	17	MeCN	0.14	0.35	0.05	**35**
**11**	DSA	17	MeCN	0.14	0.50	0.07	**36**
**12**	DSA	20	MeCN	0.20	0.35	0.07	**26**
**13**	DSA	17	$MeCN/H_2O$ (95:5)	0.14	-	-	**0**
**14**	DSA	17	$MeCN/H_2O$ (95:5)	0.14	0.35	0.05	**0**
**15**	DSA	17	$MeCN/H_2O$ (95:5)	0.14	1.00	0.14	**0**

Comment: $NBnEt_2$ was not observed in any case. Purity control of the starting material $NBnEt_3Cl$: GC yield of NEt_3 0%.

As shown in Table S1, only NEt_3 was observed, without any $NBnEt_2$. Entries 1-2 and 10-11, which showed the highest yields, were further investigated for decomposition of another electrolyte (MTBS).

Table S2: Screening of electrochemical reaction conditions for the MTBS decomposition.

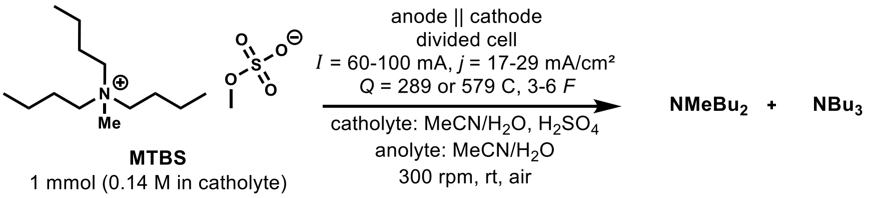

Entry	Anode	Cathode	*F*	*Q* (C)	*I* (mA)	*j* (mA/cm²)	Solvent in catholyte	$n_{H_2SO_4}$ (mmol)	$c_{H_2SO_4}$ (M)	GC YIELD NMeBu₂ (%)	GC YIELD NBu₃ (%)
**1**	Graphite	BDD	3	289	60	17	MeCN	0.35	0.05	**0**	**0**
**2**	Graphite	BDD	3	289	60	17	MeCN	0.50	0.07	**0**	**1**
**3**	Graphite	BDD	3	289	60	17	MeCN	1.00	0.14	**0**	**0**
**4**	Graphite	BDD	3	289	60	17	MeCN	2.00	0.29	**0**	**1**
**5**	Graphite	BDD	3	289	60	17	MeCN	3.00	0.43	**0**	**1**
**6**	Graphite	BDD	6	579	60	17	MeCN/H₂O (95:5)	-	-	**0**	**1**
**7**	Graphite	Ni foam	6	579	60	17	MeCN/H₂O (95:5)	-	-	**0**	**1**
**8**	Graphite	BDD	3	289	60	17	MeCN/H₂O (95:5)	-	-	**0**	**0**
**9**	Graphite	BDD	3	289	60	17	MeCN/H₂O (95:5)	0.35	0.05	**0**	**1**
**10**	Graphite	BDD	3	289	60	17	MeCN/H₂O (95:5)	0.50	0.07	**0**	**0**
**11**	Graphite	BDD	3	289	60	17	MeCN/H₂O (95:5)	1.00	0.14	**0**	**1**
**12**	Graphite	BDD	3	289	60	17	MeCN/H₂O (95:5)	2.00	0.29	**0**	**1**
**13**	Graphite	BDD	3	289	60	17	MeCN/H₂O (95:5)	3.00	0.43	**0**	**1**
**14**	Graphite	BDD	3	289	60	17	MeCN/H₂O (84:16)	-	-	**0**	**1**
**15**	Graphite	BDD	3	289	100	29	MeCN/H₂O (84:16)	-	-	**0**	**1**
**16**	DSA	BDD	3	289	60	17	MeCN	0.35	0.05	**0**	**0**
**17**	DSA	BDD	3	289	60	17	MeCN	0.50	0.07	**0**	**1**
**18**	DSA	BDD	3	289	60	17	MeCN	1.00	0.14	**0**	**0**
**19**	DSA	BDD	3	289	60	17	MeCN	3.00	0.43	**0**	**1**
**20**	DSA	BDD	3	289	60	17	MeCN	4.00	0.57	**0**	**0**
**21**	DSA	BDD	3	289	60	17	MeCN/H₂O (95:5)	-	-	**0**	**0**
**22**	DSA	BDD	3	289	60	17	MeCN/H₂O (95:5)	0.35	0.05	**0**	**0**
**23**	DSA	BDD	3	289	60	17	MeCN/H₂O (95:5)	0.50	0.07	**0**	**1**
**24**	DSA	BDD	3	289	60	17	MeCN/H₂O (95:5)	1.00	0.14	**0**	**0**
**25**	DSA	BDD	3	289	60	17	MeCN/H₂O (95:5)	2.00	0.29	**0**	**0**
**26**	DSA	BDD	3	289	60	17	MeCN/H₂O (95:5)	3.00	0.43	**0**	**0**
**27**	DSA	BDD	3	289	60	17	MeCN/H₂O (84:16)	-	-	**0**	**1**
**28**	DSA	BDD	3	289	100	29	MeCN/H₂O (84:16)	-	-	**0**	**1**
**29**	Pt	BDD	3	289	60	17	MeCN/H₂O (84:16)	-	-	**2**	**0**
**30**	Pt	BDD	3	289	100	29	MeCN/H₂O (84:16)	-	-	**2**	**1**

Comment: In cases when MeCN was used in catholyte, anolyte was dissolved in MeCN/H_2O (95:5). Purity control of the starting material MTBS: GC yield of $NMeBu_2$ is 0% and GC yield of NBu_3 is 0.55%. This value was subtracted from the measured GC yield for each reaction. In Tables S2 and S3, already calculated values are reported.

The reaction conditions for decomposition of $NBnEt_3Cl$ were applied also for MTBS, however, the results show high stability of MTBS under these conditions (entries 1-2 and 16-17 in Table S2). In contrast to $NBnEt_3Cl$, we found that the decomposition of MTBS progresses slightly better without H_2SO_4 and with the addition of H_2O.

As we got the highest yield of $NMeBu_2$ with Pt anode (entries 29 and 30 in Table S2), we continued with the screening of reaction conditions, as shown in the Table S3 below.

Table S3: Screening of electrochemical reaction conditions for the MTBS decomposition.

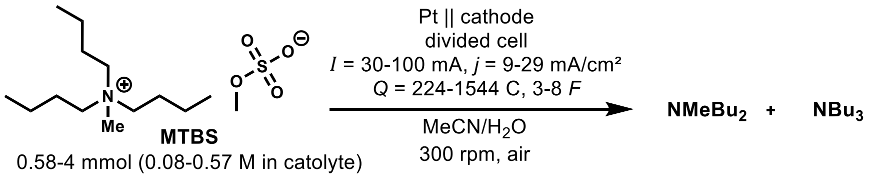

Entry	Cathode	*F*	*I* (mA)	*j* (mA/cm²)	*Q* (C)	n_{MTBS} (mmol)	c_{MTBS} (M)	MeCN/H₂O (v/v)	*T* (°C)	GC YIELD NMeBu₂ (%)	NBu₃ (%)
**1**	BDD	3	100	29	289	1.00	0.14	84:16	rt	**2**	**1**
**2**	BDD	3	60	17	289	1.00	0.14	84:16	rt	**2**	**0**
**3**	BDD	4	100	29	386	1.00	0.14	84:16	rt	**2**	**1**
**4**	BDD	4	60	17	386	1.00	0.14	84:16	rt	**3**	**1**
**5**	BDD	4	30	9	386	1.00	0.14	84:16	rt	**4**	**1**
**6**	BDD	4	30	9	386	1.00	0.14	95:5	rt	**2**	**0**
**7**	BDD	4	100	29	224	0.58	0.08	84:16	rt	**1**	**0**
**8**	BDD	4	100	29	772	2.00	0.29	84:16	rt	**5**	**1**
**9**	BDD	4	100	29	1158	3.00	0.43	84:16	rt	**4**	**0**
**10**	BDD	4	100	29	1158	3.00	0.43	84:16	50	**6**	**0**
**11**	BDD	4	100	29	1544	4.00	0.57	84:16	rt	**4**	**0**
**12**	BDD	8	100	29	772	1.00	0.14	84:16	rt	**3**	**1**
**13**	BDD	8	60	17	772	1.00	0.14	84:16	rt	**2**	**0**
**14**	BDD	8	60	17	772	1.00	0.14	84:16	50	**3**	**0**
**15**	BDD	8	30	9	772	1.00	0.14	84:16	rt	**3**	**1**
**16**	BDD	8	60	17	1544	2.00	0.29	84:16	rt	**4**	**0**
**17**	BDD	8	60	17	1544	2.00	0.29	84:16	50	**5**	**1**
**18**	graphite	4	100	29	224	0.58	0.08	84:16	rt	**1**	**0**
**19**	GC	4	100	29	224	0.58	0.08	84:16	rt	**1**	**0**
**20**	Cu	4	100	29	224	0.58	0.08	84:16	rt	**2**	**0**
**21**	Cu	8	60	17	772	1.00	0.14	84:16	rt	**2**	**0**
**22**	Pb	4	30	9	386	1.00	0.14	84:16	rt	**5**	**1**

Comment: GC is glassy carbon. Purity control of the starting material MTBS: GC yield of $NMeBu_2$ is 0% and GC yield of NBu_3 is 0.55%. This value was subtracted from the measured GC yield for each reaction. In Tables S2 and S3, already calculated values are reported.

With conditions in entries 5 and 22 (Table S3), we continued our investigation. These entries were selected because they showed the highest degree of decomposition in the shortest time for 1 mmol of the starting material.

Table S4: Screening of electrochemical reaction conditions for the decomposition of alkylammonium salt.

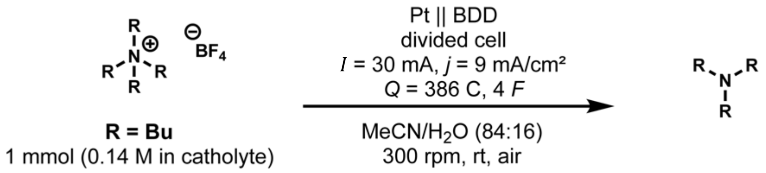

Entry	R	GC yield NR₃ (%)
**1**	Bu	**3**

Comment: Purity control of starting material NBu_4BF_4: GC yield of NBu_3 0%.

Table S5: Screening of electrochemical reaction conditions for the decomposition of 1-butyl-1-methyl-pyrrolidinium bis(trifluoromethylsulfonyl)imide.

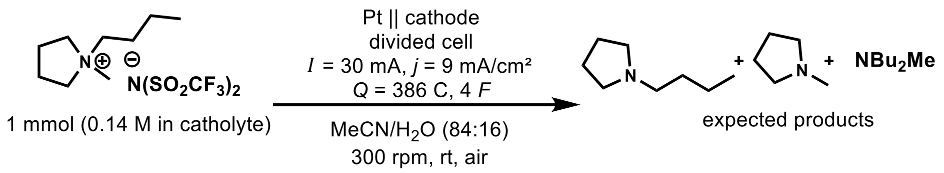

Entry	cathode	GC yield (%)
**1**	BDD	**0**
**2**	Pb	**0**

2 Computational Details

2.1 Computing Hardware

Parts of this research were conducted using the supercomputer MOGON 2 and advisory services offered by Johannes Gutenberg University Mainz (hpc.uni-mainz.de), which is a member of the AHRP (Alliance for High Performance Computing in Rhineland Palatinate, www.ahrp.info) and the Gauss Alliance e.V.

2.2 Dataset Preparation

For the calculations and subsequent analysis, we utilized the dataset described in Ref.[S3], which comprises 6650 quaternary ammonium cations along with their associated properties. The dataset was created by filtering the PubChem database[S4] for molecules including NR_4^+ fragments, with cations with charges higher than +4 and molar mass greater than 500 g/mol being excluded. In the case of the cations including other atoms than carbon, hydrogen and nitrogen, we limited the unbranched chain length to 6 atoms. The initial molecular structures were directly taken from the PubChem database if available, otherwise they were constructed from the appropriate SMILES[S5] codes. Each cation of the dataset was then processed in a quantum chemical workflow as described in the main text and below.

To ensure reliable discussion of the reduction and oxidation behavior, it was essential to work with well-converged structures of the cations as well as their oxidized and reduced counterparts. Therefore, the original dataset was refined by excluding structures where the calculated harmonic frequencies for the oxidized or reduced species exhibited imaginary components, which could signify a transition state rather than a stable equilibrium structure. Such structures were initially allowed, whereas the cations itself were required to be in an equilibrium state from the start on. Filtering the dataset in that way, a set of 5392 quaternary ammonium cations remained.

2.3 Feature Calculations

For each cation, the following computational protocol was employed:

Initial conformational sampling was carried out using the *Crest*[S5,6] program (version 2.12) at the GFN2-xTB[S7] level of theory. The resulting lowest-energy conformer was used to perform a two-step geometry optimization. In the first step, a pre-optimization was performed using the *xTB* program[S8] (version 6.5.1) at the GFN2-xTB-niveau with a convergence criterium of 1×10^{-8} Hartree for the energy change in the SCF cycle, 5×10^{-8} Hartree for the energy change in the geometry optimization and 5×10^{-5} Hartree/Bohr for the gradient. The second step involved a more refined geometry optimization using the *ORCA* program[S9,10] (version 5.0.3) with the B97-3c composite method[S11]. SCF convergence thresholds were set to 1×10^{-8} Hartree for the energy change and 1×10^{-7}a.u. for the density change. These settings were enforced via the *TightSCF* keyword. For the geometry optimization itself, convergence criteria of 5×10^{-6} Hartree for the energy, 1×10^{-4} Hartree/Bohr for the gradient, and 2×10^{-3} Bohr for the coordinate change were applied. The resolution of the identity (RI) approximation was employed as implemented in ORCA[S12] using an appropriate auxiliary basis set.[S13] Integration of the exchange-correlation potential was performed using the *DEFGRID2* integration grid.[S14] In cases where convergence issues arose, the *SlowConv* keyword was included. If problems persisted, *VerySlowConv* was used as a more robust alternative.

Thermodynamic properties were calculated following the same methodology as outlined above. Additionally, the *AnFreq* keyword was employed to initiate analytical frequency calculations, which form the basis for all derived thermodynamic quantities.

Solvation energies were computed using the COSMO-RS model[S15,16] as implemented in the *COSMOtherm* program[S17] (version 2021) with the BP_TZVPD_FINE_21.ctd parametrization. Solvent data for acetonitrile were taken from the COSMOtherm database. For the cation, single-point calculations were conducted using the *TURBOMOLE* program[S18,19] (version 6.5) to obtain both the gas-phase energy and the COSMO[S20,21] cavity surface. These calculations were performed at the DFT level[S22] using the BP86 functional[S23,24] and the def2-TZVPD basis set[S25], applied to the ORCA-optimized geometry. For integration of the exchange correlation potential the m4 grid was employed.[S26] Also, the RI approximation[S26–28] with the def2-TZVP auxiliary basis set[S13] as well as the multipole approximation (MARI-J)[S29] were used. The convergence criterium for the energy in the SCF cycle was set to 1×10^{-6} hartree. In cases of SCF convergence difficulties, damping techniques were applied, with parameter sets adapted to the specific situation.

The same computational protocol was used for the reduced and oxidized species. Their initial geometries were derived from the optimized cationic structures. For the reduced species, significant structural deviations were often observed, prompting an additional conformational search using *Crest* to identify a suitable starting geometry.

The calculation of the reorganization energy required two additional calculation steps to obtain the energy of the cation at its reduced structure and vice versa. These calculations were also performed using *ORCA* with the same settings as previously described.

The reduction potential for the ferrocenium/ferrocene redox couple was calculated in the same way as for the cations.

2.4 Benchmark Calculations

To validate the results of the calculations using the B97-3c composite method and justify their physical relevance, benchmark calculations for a subset of 100 cations of the initial dataset were performed. For this, in principle the same procedure was conducted as described before. Instead of the B97-3c method, the range-separated hybrid functional ωB97M-V[S30] from the LibXC library[S31] was employed in combination with the def2-TZVPPD[S25,32] triple zeta basis set. All other settings were kept, but the calculation of the harmonic frequencies had to be performed numerically, as the analytical second derivatives were not implemented for this functional.

2.5 MD Simulation Details

The MD simulations were performed with the following settings:

The simulations were performed using the GROMACS program package[S33,34] (version 2021.3), the OPLS-AA force field[S35,36] and the LEAPFROG algorithm.[S37]

All bonds containing hydrogen were constrained using the LINCS algorithm,[S38] allowing for a timestep of 2 fs. We used a cut-off of 1.0 nm for all short-range interactions. The long-range Coulomb interactions were computed employing the particle mesh Ewald summation method[S39] and for the van der Waals interaction we applied a standard dispersion correction.[S40] All simulations were performed using periodic boundary conditions and the neighbor list was updated every 20 fs. After an energy minimization, a thermalization at 298 K using a velocity rescaling thermostat[S41] with a time constant of 0.1 ps was performed. This was followed by a NPT equilibration via coupling to a Berendsen barostat[S42] with a time constant of 2 ps and a pressure of 1 bar. The compressibilities were set to 9.6×10^{-5} bar^{-1} for MeCN and to 9.7×10^{-5} bar^{-1} for THF. All production runs were performed using the systems prepared this way.

3 Benchmark Results

To validate the results obtained with the B97-3c method, we performed benchmark calculations on a subset of 100 quaternary ammonium cations from our initial dataset using the more advanced ωB97M-V functional. Figure S4 illustrates the correlation between the reduction and oxidation potentials calculated with both methods, allowing for a direct assessment of their relative accuracy with respect to these properties.

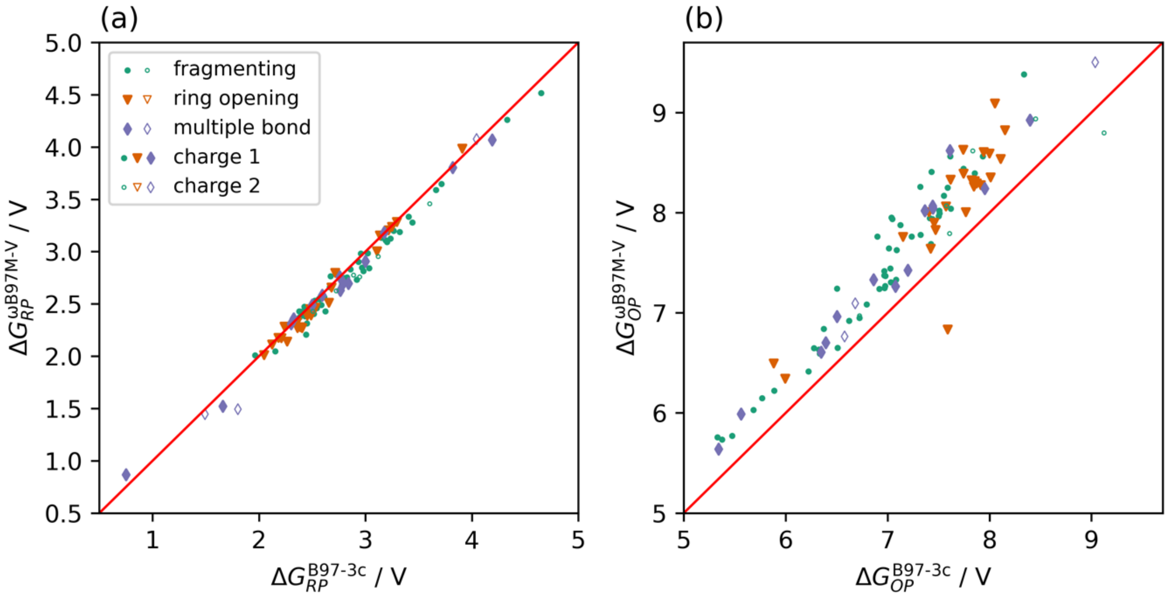

Figure S4: Comparison of reduction potentials (a) and oxidation potentials (b) calculated using both methods described in section 2 for a subset of 100 quaternary ammonium cations.

While the reduction potentials are largely consistent between the two methods, the oxidation potentials predicted by the ωB97M-V functional are systematically shifted to on average about 0.5 V higher values. Nevertheless, the overall trend observed with the B97-3c method is retained.

Given that the ωB97M-V functional is a hybrid functional incorporating a portion of exact exchange, in contrast to the B97-3c method, one might expect a stronger correlation between reduction potentials and LUMO energies, as well as oxidation potentials and HOMO energies. Figure S5 presents these correlation plots side by side for both methods. Panels (a) and (b) clearly show that no statistically significant differences arise between the methods in this regard. Similarly, the degree of correlation observed in panels (c) and (d) is nearly identical. From this perspective, neither method demonstrates clear superiority over the other.

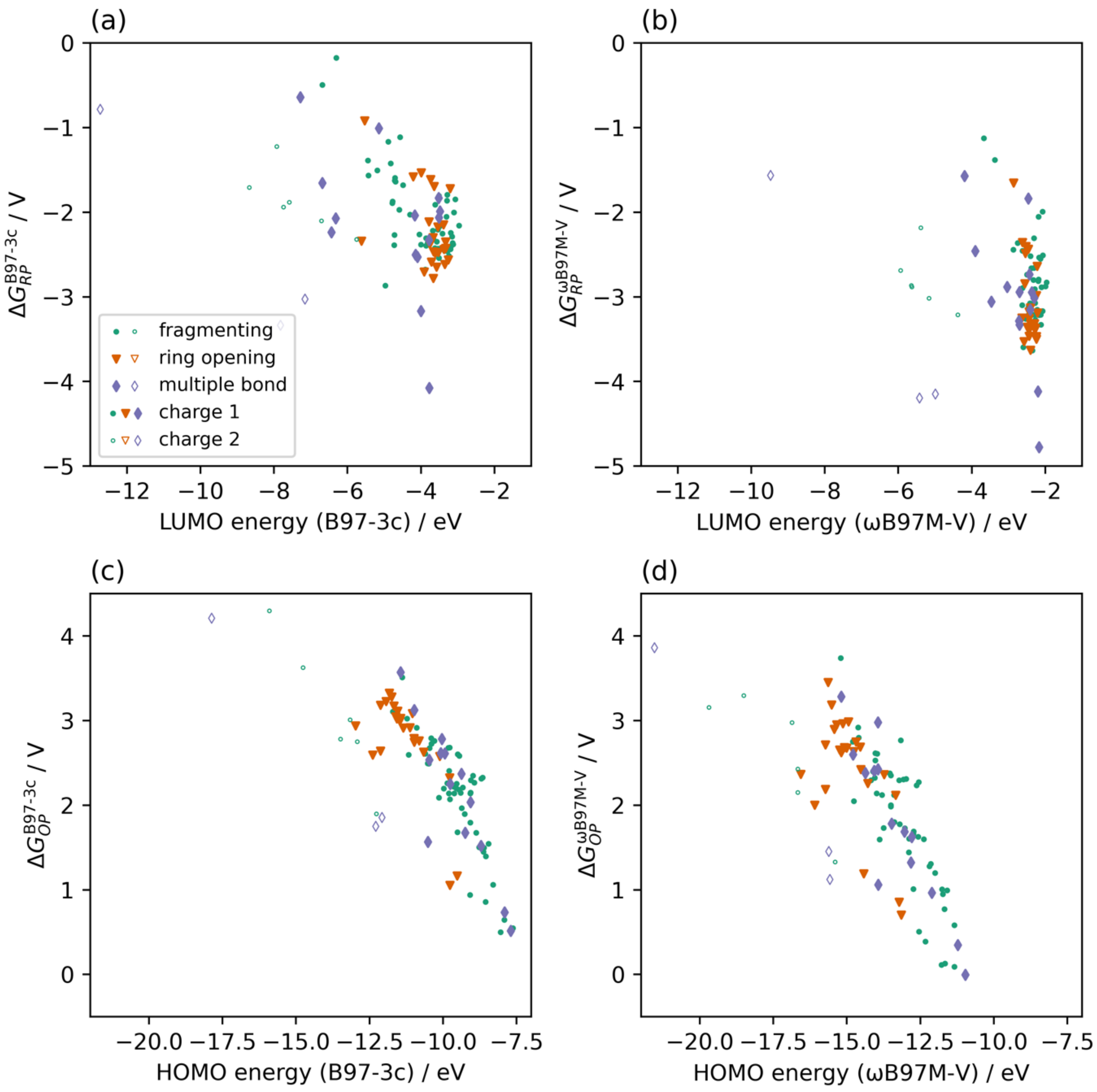

Figure S5: Comparison of correlations between reduction potential and LUMO energy in (a) and (b) and between oxidation potential and HOMO energy in (c) and (d), calculated by using two different methods. In (a) and (c) the B97-3c method was employed, whereas in (b) and (d) the ωB97M-V/def2-TZVPPD method was utilized.

As shown in Figure S6, the trends observed for the redox potentials also apply to the inner-sphere reorganization energies. For each cation, the values obtained with both methods are in close agreement. Overall, the data points exhibit slightly greater scatter around the diagonal, with the ωB97M-V functional tending to yield marginally higher values on average.

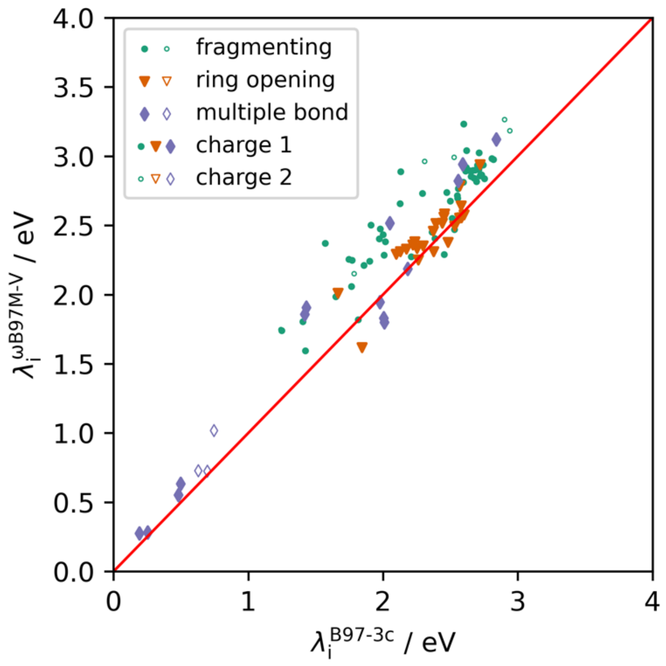

Figure S6: Comparison of inner-sphere reorganization energies calculated employing both methods described in section 2.

4 Data and Materials Availability

The dataset discussed in section 2 and 3 of this document is published in a repository on Zenodo.

DOI: https://doi.org/10.5281/zenodo.14288293

5 References

(S1) Armarego, W. L. F.; Chai, C. L. L. *Purification of Laboratory Chemicals*, 7th ed.; Elsevier/Butterworth-Heinemann: Amsterdam London, 2013.

(S2) Gütz, C.; Klöckner, B.; Waldvogel, S. R. Electrochemical Screening for Electroorganic Synthesis. *Org. Process Res. Dev.* **2016**, *20* (1), 26–32.

(S3) Mast, F.; Hielscher, M. M.; Wirtanen, T.; Erichsen, M.; Gauss, J.; Diezemann, G.; Waldvogel, S. R. Choice of the Right Supporting Electrolyte in Electrochemical Reductions: A Principal Component Analysis. *J. Am. Chem. Soc.* **2024**, *146* (22), 15119–15129.

(S4) Kim, S.; Chen, J.; Cheng, T.; Gindulyte, A.; He, J.; He, S.; Li, Q.; Shoemaker, B. A.; Thiessen, P. A.; Yu, B.; Zaslavsky, L.; Zhang, J.; Bolton, E. E. PubChem 2023 Update. *Nucleic Acids Res.* **2023**, *51* (D1), D1373–D1380.

(S5) Pracht, P.; Grimme, S.; Bannwarth, C.; Bohle, F.; Ehlert, S.; Feldmann, G.; Gorges, J.; Müller, M.; Neudecker, T.; Plett, C.; Spicher, S.; Steinbach, P.; Wesołowski, P. A.; Zeller, F. CREST—a Program for the Exploration of Low-Energy Molecular Chemical Space. *J. Chem. Phys.* **2024**, *160* (11), 114110.

(S6) Pracht, P.; Bohle, F.; Grimme, S. Automated Exploration of the Low-Energy Chemical Space with Fast Quantum Chemical Methods. *Phys. Chem. Chem. Phys.* **2020**, *22* (14), 7169–7192.

(S7) Bannwarth, C.; Ehlert, S.; Grimme, S. GFN2-xTB—an Accurate and Broadly Parametrized Self-Consistent Tight-Binding Quantum Chemical Method with Multipole Electrostatics and Density-Dependent Dispersion Contributions. *J. Chem. Theory Comput.* **2019**, *15* (3), 1652–1671.

(S8) Bannwarth, C.; Caldeweyher, E.; Ehlert, S.; Hansen, A.; Pracht, P.; Seibert, J.; Spicher, S.; Grimme, S. Extended TIGHT-BINDING Quantum Chemistry Methods. *WIREs Comput. Mol. Sci.* **2021**, *11* (2), e1493.

(S9) Neese, F. The ORCA Program System. *WIREs Comput. Mol. Sci.* **2012**, *2* (1), 73–78.

(S10) Neese, F.; Wennmohs, F.; Becker, U.; Riplinger, C. The ORCA Quantum Chemistry Program Package. *J. Chem. Phys.* **2020**, *152* (22), 224108.

(S11) Brandenburg, J. G.; Bannwarth, C.; Hansen, A.; Grimme, S. B97-3c: A Revised Low-Cost Variant of the B97-D Density Functional Method. *J. Chem. Phys.* **2018**, *148* (6), 64104.

(S12) Neese, F. An Improvement of the Resolution of the Identity Approximation for the Formation of the Coulomb Matrix. *J. Comput. Chem.* **2003**, *24* (14), 1740–1747.

(S13) Weigend, F. Accurate Coulomb-Fitting Basis Sets for H to Rn. *Phys. Chem. Chem. Phys.* **2006**, *8* (9), 1057.

(S14) Helmich-Paris, B.; de Souza, B.; Neese, F.; Izsák, R. An Improved Chain of Spheres for Exchange Algorithm. *J. Chem. Phys.* **2021**, *155* (10), 104109.

(S15) Klamt, A. Conductor-like Screening Model for Real Solvents: A New Approach to the Quantitative Calculation of Solvation Phenomena. *J. Phys. Chem.* **1995**, *99* (7), 2224–2235.

(S16) Klamt, A.; Jonas, V.; Bürger, T.; Lohrenz, J. C. W. Refinement and Parametrization of COSMO-RS. *J. Phys. Chem. A* **1998**, *102* (26), 5074–5085.

(S17) DassaultSystemes. BIOVIA COSMOtherm: The Leading COSMO-RS Application in Solvation Chemistry, 2021. http://www.3ds.com.

(S18) Ahlrichs, R.; Bär, M.; Häser, M.; Horn, H.; Kölmel, C. Electronic Structure Calculations on Workstation Computers: The Program System Turbomole. *Chem. Phys. Lett.* **1989**, *162* (3), 165–169.

(S19) Balasubramani, S. G.; Chen, G. P.; Coriani, S.; Diedenhofen, M.; Frank, M. S.; Franzke, Y. J.; Furche, F.; Grotjahn, R.; Harding, M. E.; Hättig, C.; Hellweg, A.; Helmich-Paris, B.; Holzer, C.; Huniar, U.; Kaupp, M.; Marefat Khah, A.; Karbalaei Khani, S.; Müller, T.; Mack, F.; Nguyen, B. D.; Parker, S. M.; Perlt, E.; Rappoport, D.; Reiter, K.; Roy, S.; Rückert, M.; Schmitz, G.; Sierka, M.; Tapavicza, E.; Tew, D. P.; Van Wüllen, C.; Voora, V. K.; Weigend, F.; Wodyński, A.; Yu, J. M. TURBOMOLE: Modular Program Suite for Ab Initio Quantum-Chemical and Condensed-Matter Simulations. *J. Chem. Phys.* **2020**, *152* (18), 184107.

(S20) Klamt, A.; Schüürmann, G. COSMO: A New Approach to Dielectric Screening in Solvents with Explicit Expressions for the Screening Energy and Its Gradient. *J. Chem. Soc., Perkin Trans. 2* **1993**, No. 5, 799–805.

(S21) Schäfer, A.; Klamt, A.; Sattel, D.; Lohrenz, J. C. W.; Eckert, F. COSMO Implementation in TURBOMOLE: Extension of an Efficient Quantum Chemical Code towards Liquid Systems. *Phys. Chem. Chem. Phys.* **2000**, *2* (10), 2187–2193.

(S22) Treutler, O.; Ahlrichs, R. Efficient Molecular Numerical Integration Schemes. *J. Chem. Phys.* **1995**, *102* (1), 346–354.

(S23) Becke, A. D. Density-Functional Exchange-Energy Approximation with Correct Asymptotic Behavior. *Phys. Rev. A* **1988**, *38* (6), 3098–3100.

(S24) Perdew, J. P. Density-Functional Approximation for the Correlation Energy of the Inhomogeneous Electron Gas. *Phys. Rev. B* **1986**, *33* (12), 8822–8824.

(S25) Rappoport, D.; Furche, F. Property-Optimized Gaussian Basis Sets for Molecular Response Calculations. *J. Chem. Phys.* **2010**, *133* (13), 134105.

(S26) Eichkorn, K.; Weigend, F.; Treutler, O.; Ahlrichs, R. Auxiliary Basis Sets for Main Row Atoms and Transition Metals and Their Use to Approximate Coulomb Potentials. *Theor. Chem. Acta* **1997**, *97* (1–4), 119–124.

(S27) Eichkorn, K.; Treutler, O.; Öhm, H.; Häser, M.; Ahlrichs, R. Auxiliary Basis Sets to Approximate Coulomb Potentials (Chem. Phys. Letters 240 (1995) 283-290). *Chem. Phys. Lett.* **1995**, *242* (6), 652–660.

(S28) Weigend, F. A Fully Direct RI-HF Algorithm: Implementation, Optimised Auxiliary Basis Sets, Demonstration of Accuracy and Efficiency. *Phys. Chem. Chem. Phys.* **2002**, *4* (18), 4285–4291.

(S29) Sierka, M.; Hogekamp, A.; Ahlrichs, R. Fast Evaluation of the Coulomb Potential for Electron Densities Using Multipole Accelerated Resolution of Identity Approximation. *J. Chem. Phys.* **2003**, *118* (20), 9136–9148.

(S30) Mardirossian, N.; Head-Gordon, M. ωB97M-V: A Combinatorially Optimized, Range-Separated Hybrid, Meta-GGA Density Functional with VV10 Nonlocal Correlation. *J. Chem. Phys.* **2016**, *144* (21), 214110.

(S31) Lehtola, S.; Steigemann, C.; Oliveira, M. J. T.; Marques, M. A. L. Recent Developments in Libxc — a Comprehensive Library of Functionals for Density Functional Theory. *Softwarex* **2018**, *7*, 1–5.

(S32) Weigend, F.; Ahlrichs, R. Balanced Basis Sets of Split Valence, Triple Zeta Valence and Quadruple Zeta Valence Quality for H to Rn: Design and Assessment of Accuracy. *Phys. Chem. Chem. Phys.* **2005**, *7* (18), 3297.

(S33) Bekker, H.; Berendsen, H.; Dijkstra, E. J.; Achterop, S.; Drunen, R.; van der Spoel, D.; Sijbers, A.; Keegstra, H.; Reitsma, B.; Renardus, M. K. R. Gromacs: A Parallel Computer for Molecular Dynamics Simulations. In *Physics Computing*; 1993; Vol. 92, pp 252–256.

(S34) Abraham, M. J.; Murtola, T.; Schulz, R.; Páll, S.; Smith, J. C.; Hess, B.; Lindahl, E. GROMACS: High Performance Molecular Simulations through Multi-Level Parallelism from Laptops to Supercomputers. *SoftwareX* **2015**, *1–2*, 19–25.

(S35) Jorgensen, W. L.; Tirado-Rives, J. The OPLS [Optimized Potentials for Liquid Simulations] Potential Functions for Proteins, Energy Minimizations for Crystals of Cyclic Peptides and Crambin. *J. Am. Chem. Soc.* **1988**, *110* (6), 1657–1666.

(S36) Jorgensen, W. L.; Maxwell, D. S.; Tirado-Rives, J. Development and Testing of the OPLS All-Atom Force Field on Conformational Energetics and Properties of Organic Liquids. *J. Am. Chem. Soc.* **1996**, *118* (45), 11225–11236.

(S37) Hockney, R. W.; Eastwood, J. W. *Computer Simulation Using Particles*, Special student ed.; A. Hilger: Bristol [England] ; Philadelphia, 1988.

(S38) Hess, B.; Bekker, H.; Berendsen, H. J. C.; Fraaije, J. G. E. M. LINCS: A Linear Constraint Solver for Molecular Simulations. *J. Comput. Chem.* **1997**, *18* (12), 1463–1472.

(S39) Darden, T.; York, D.; Pedersen, L. Particle Mesh Ewald: An N·log(N) Method for Ewald Sums in Large Systems. *J. Chem. Phys.* **1993**, *98* (12), 10089–10092.

(S40) Allen, M. P.; Tildesley, D. J. *Computer Simulation of Liquids*, 2nd ed.; Oxford university press: Oxford, 2017.

(S41) Bussi, G.; Donadio, D.; Parrinello, M. Canonical Sampling through Velocity Rescaling. *J. Chem. Phys.* **2007**, *126* (1), 14101.

(S42) Berendsen, H. J. C.; Postma, J. P. M.; Van Gunsteren, W. F.; DiNola, A.; Haak, J. R. Molecular Dynamics with Coupling to an External Bath. *J. Chem. Phys.* **1984**, *81* (8), 3684–3690.
